# Remotely Sensed Data Informs Red List Evaluations and Conservation Priorities in Southeast Asia

**DOI:** 10.1371/journal.pone.0160566

**Published:** 2016-08-03

**Authors:** Binbin V. Li, Alice C. Hughes, Clinton N. Jenkins, Natalia Ocampo-Peñuela, Stuart L. Pimm

**Affiliations:** 1 Nicholas School of the Environment, Duke University, Durham, North Carolina, United States of America; 2 Center for Integrative Conservation, Xishuangbanna Tropical Botanical Gardens, Chinese Academy of Sciences, Menglun, Yunnan, China; 3 Instituto de Pesquisas Ecológicas, Nazaré Paulista, São Paulo, Brazil; Clemson University, UNITED STATES

## Abstract

The IUCN Red List has assessed the global distributions of the majority of the world’s amphibians, birds and mammals. Yet these assessments lack explicit reference to widely available, remotely-sensed data that can sensibly inform a species’ risk of extinction. Our first goal is to add additional quantitative data to the existing standardised process that IUCN employs. Secondly, we ask: do our results suggest species of concern—those at considerably greater risk than hitherto appreciated? Thirdly, these assessments are not only important on a species-by-species basis. By combining distributions of species of concern, we map conservation priorities. We ask to what degree these areas are currently protected and how might knowledge from remote sensing modify the priorities? Finally, we develop a quick and simple method to identify and modify the priority setting in a landscape where natural habitats are disappearing rapidly and so where conventional species’ assessments might be too slow to respond. Tropical, mainland Southeast Asia is under exceptional threat, yet relatively poorly known. Here, additional quantitative measures may be particularly helpful. This region contains over 122, 183, and 214 endemic mammals, birds, and amphibians, respectively, of which the IUCN considers 37, 21, and 37 threatened. When corrected for the amount of remaining natural habitats within the known elevation preferences of species, the average sizes of species ranges shrink to <40% of their published ranges. Some 79 mammal, 49 bird, and 184 amphibian ranges are <20,000km^2^—an area at which IUCN considers most other species to be threatened. Moreover, these species are not better protected by the existing network of protected areas than are species that IUCN accepts as threatened. Simply, there appear to be considerably more species at risk than hitherto appreciated. Furthermore, incorporating remote sensing data showing where habitat loss is prevalent changes the locations of conservation priorities.

## Introduction

The IUCN Red List [1] classifies species into various categories that reflect their risk of extinction. It employs rigorously objective criteria, is transparent, and democratic in soliciting comments on individual species decisions. It aspires to provide a global analysis of the status and distributions of all species based on the best available data and expert analysis for each species. The process updates many species regularly, and the associated data are publicly accessible. As individuals who contribute to these assessments and encourage species-specific research to address limited data, we nonetheless worry that important data do not enter this process as effectively as they might. In particular, the rapid growth in remote sensing provides geographically fine-scale data on elevations and increasingly sophisticated characterisations of land use and land use change [2]. Moreover, there are global databases on which areas are protected—the first line of defence at ensuring species survival [3, 4].

There are two specific challenges. First, there are taxa and areas that are poorly known, where species distributions are incomplete and out of date, and for which the Red List considers many assessments as data deficient. Second, in regions where human actions are rapidly destroying natural habitats, conventional species’ assessments might be too slow to respond.

What quantitative measures can we derive from widely available, remotely sensed data that sensibly inform a species’ risk of extinction? Some of these inputs may informally contribute to existing Red List determinations, but we show that we can succinctly and easily include additional quantitative data for each species in a standardised way. Our goal is not to replace the existing process, nor even subject it to unwarranted criticism, but to add additional tools to the process. These add precision and accuracy that until recently were impossible, and which help accurately assess species true distribution and status. Secondly, will our results suggest species of concern—those at considerably greater risk than hitherto appreciated? These assessments are not only important on a species-by-species basis, but by combining distributions of threatened species, we create maps of conservation priorities and assay to what degree these areas are currently protected. Thirdly, we ask how might knowledge from remote sensing modify the priorities? Finally, can we develop a quick and simple method to identify and modify the priority setting in a landscape where human actions are rapidly expanding?

We wish to ask these questions in a region that is under exceptional threat, yet relatively poorly known. For such a region, additional quantitative measures may be particularly helpful. Thus, we consider the birds, mammals, and amphibians of tropical, mainland Southeast Asia. We define this region to be from the province of Yunnan, China, to eastern India, and south to Singapore. The study area encompasses 2,691,773 km^2^ and is in the heart of the Indo-Burma Biodiversity Hotspot and northern Sundaland Hotspot [5]. We further restricted consideration to those species that are broadly endemic to this region, meaning that >80% of each species’ range occurred there. Endemism has always been one of the key targets for setting priorities, [6]. Small ranged species are much more likely to be at risk of extinction [7]. Furthermore, restricted range species tend to be locally rare within those ranges [8], further increasing a species’ risk of extinction.

There are several reasons to choose these taxa in this region. First, these species form a substantial sample and so a case study for tropical areas globally. This area also has many endemic species [9], and such species are more at risk of extinction than widespread ones [2, 5, 7, 10, 11]. This region also has many species that IUCN considers to be data deficient [12]. We must not only ask if we can identify species with unappreciated risks of extinction but assay if remotely sensed data can aid the threat assessments of species for which we otherwise know too little.

This Southeast Asian region suffers rapid deforestation—the major driving force behind global biodiversity loss [13]. It had 0.48 million ha per year net deforestation from 2000–2010, twice the rate from 1990–2000 [14], and is increasing [15]. Thus, traditional species-by-species determinations of risk may be too slow to recognise constantly changing threats. Furthermore, the deforestation often occurs as conversion of natural forests to commercial tree plantations of rubber, oil palm, and other trees crops [16, 17]. Mainland Southeast Asian countries account for 56% of the global rubber production and 39% of the palm oil production [18]. Much of the expansion of these plantations occurs at the cost of natural forests and peatlands [16, 19, 20].

The majority of agricultural and forestry expansions are monocultures rather than the traditional mixed agroforestry systems [21]. These plantations support few species [22, 23] and so have potentially catastrophic biodiversity impacts [24–26]. Unfortunately, rubber and oil palm plantations pose a particular challenge to identify and map at broad geographical scales from remote sensing. In traditional forms of classification they are almost indistinguishable from natural forests in their spectral reflectance characteristics [27], and are hard to differentiate from sparse or degraded forest when they are young [27]. Although regional efforts have identified these tree plantations [27, 28], most of the global datasets do not differentiate them in forest classifications [29].

Finally, this is a fortuitous time to ask these questions. Data on vertebrate species ranges, fine-scale topography, and protected areas have been readily available for some years. Fine-scale tree cover and plantation data, both essential to our estimates of remaining habitat, have only been available since 2014 and 2015 respectively. We expect these datasets to be improved and updated periodically, as satellite technology and classification algorithms continue to be enhanced. The approaches outlined here are designed to adapt to such changes and integrate them into further analyses.

Our methods follow a sequential process that starts from the available species range maps[1]. We have applied similar methods to address related questions about other priority areas for biodiversity—Central America[30], coastal Brazil [31], the Western Andes of Colombia [32], the USA [33], and China [34]. The first step recognises that in mountainous regions, such as here, the elevational limits of species reduce the actual habitat below what the typical range-wide maps show—and often by an order of magnitude. Moreover, the nature of montane landscapes is such that actual ranges are also substantially fragmented. Next, to the extent possible, we further refine species’ ranges by the amount of remaining habitat. We consider species in this region that are forest-dependent, because they are the majority of species and forests are relatively easy to identify compared to other land cover types like shrublands or grasslands.

## Methods

### Study area and species

**Fig 1:**
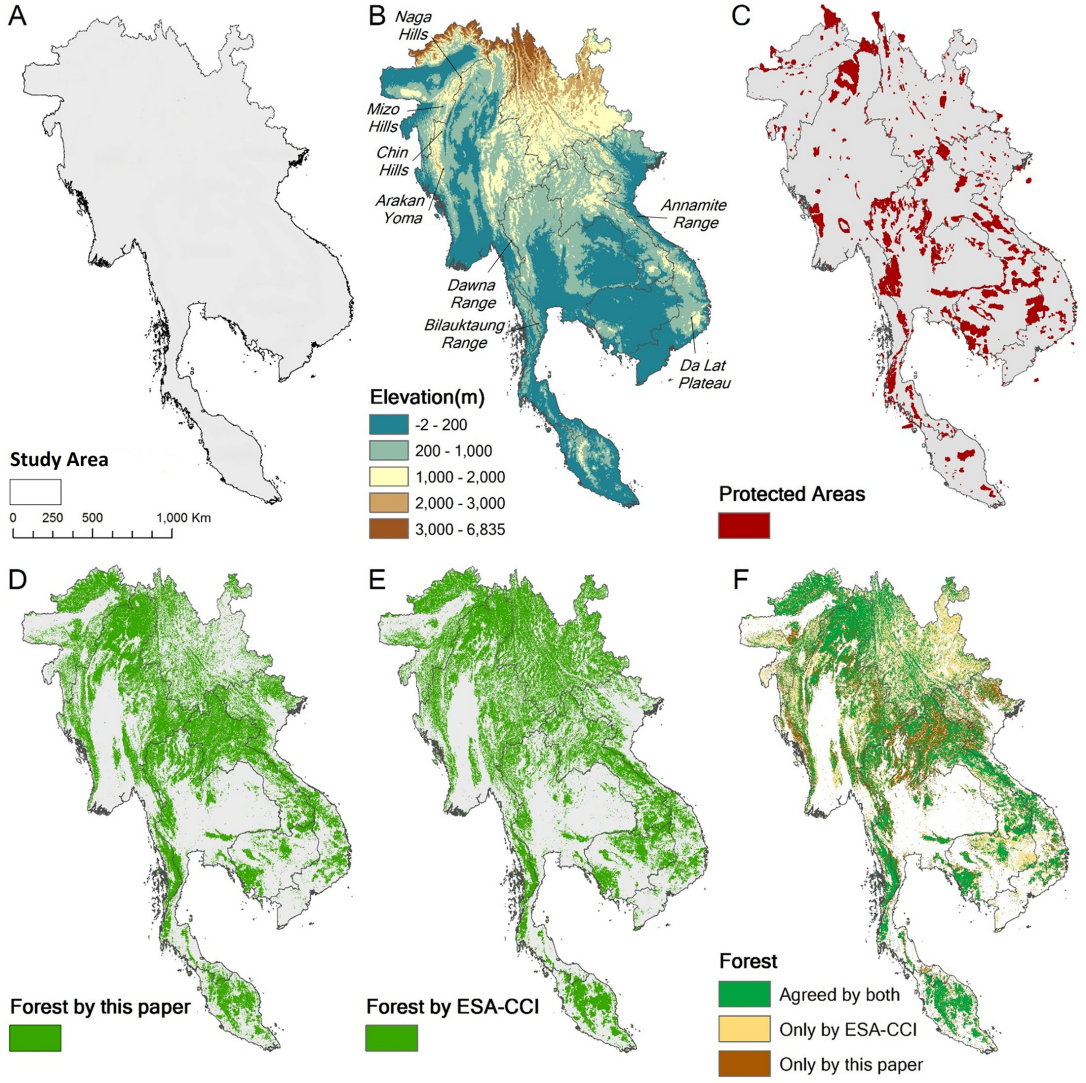
Study area. A) Boundary of the study area. B) Elevation with names of major mountain ranges. C) Protected Areas from WDPA. D) Forest cover according to our definition. E) Forest classified by ESA. F) Comparison between the two forest maps.

The study area includes Yunnan Province of China, Arunachal Pradesh, six eastern states of India (Assam, Manipur, Meghalaya, Mizoram, Nagaland, and Tripura), Laos, Vietnam, Thailand, Cambodia, Myanmar, Peninsular Malaysia, and Singapore (Fig 1A). We defined the species endemic to this area as those that have more than 80% of their range (breeding range for birds) within it. We compiled the species lists and range maps for terrestrial mammals and amphibians from the IUCN [1], and for birds from Birdlife International [35]. In total, 209 birds, 165 mammals, and 286 amphibians were endemic to this region. Among them, the IUCN has classified 30 birds, 43 mammals, and 40 amphibians as being threatened: critically endangered, endangered, or vulnerable.

### Elevational range and suitable habitat

We compiled elevational ranges and habitat preferences from IUCN for mammals and amphibians, and from [36] for birds. To be conservative and reduce bias caused by the few records of some species, we applied a minimum of 500m elevational width. If a species’ elevational range fell below this width or only one listed elevation was available, we took the midpoint and extended 250m on both sides. As the lower end does not extend below sea level, the lowest elevational range was set to 0-500m. Some species did not have enough information for their elevational ranges. If the only available information was that a species occurred in “lowland” or “montane,” we used 0-1000m and >1000m respectively. If there was no information, we did not put a limitation on its elevational range.

We compiled habitat information from the classification schemes in individual species accounts in available databases[1, 36]. Only habitats deemed suitable in published accounts were included. We defined forest species as those that only depend on forests, as well as mammal species that depend on both forest and caves, and amphibians that depend on both forest and wetland. In total, we included 183 birds, 122 mammals, and 213 amphibians as forest species. Among them, 21 birds, 37 mammals, and 37 amphibians are currently listed as threatened species and a further 39 mammals and 111 amphibians are listed as data deficient.

### Forest

Southeast Asia has a range of forest types, ranging from relatively sparse dry forests to dense rainforests. We obtained data on continuous tree-cover for 2005 [37] for the entire region. We then reclassified the percent tree cover to produce four forest classifications that used 30, 40, 50, and 60 percent cover as the cut-off for whether we consider an area forested or not.

Sexton et al. (2013) show the forest cover of ten years ago, and since then there have been major changes. We updated these forest cover estimates using Hansen's forest loss data [38] that shows deforestation since 2005. We further excluded the areas that Li and Fox [27] identified as rubber plantations and areas classified as oil palm plantations from Miettinen et al.'s study [39]. Outputs from these steps are forest layers showing 30%, 40%, 50%, and 60% forest cover.

**Fig 2:**
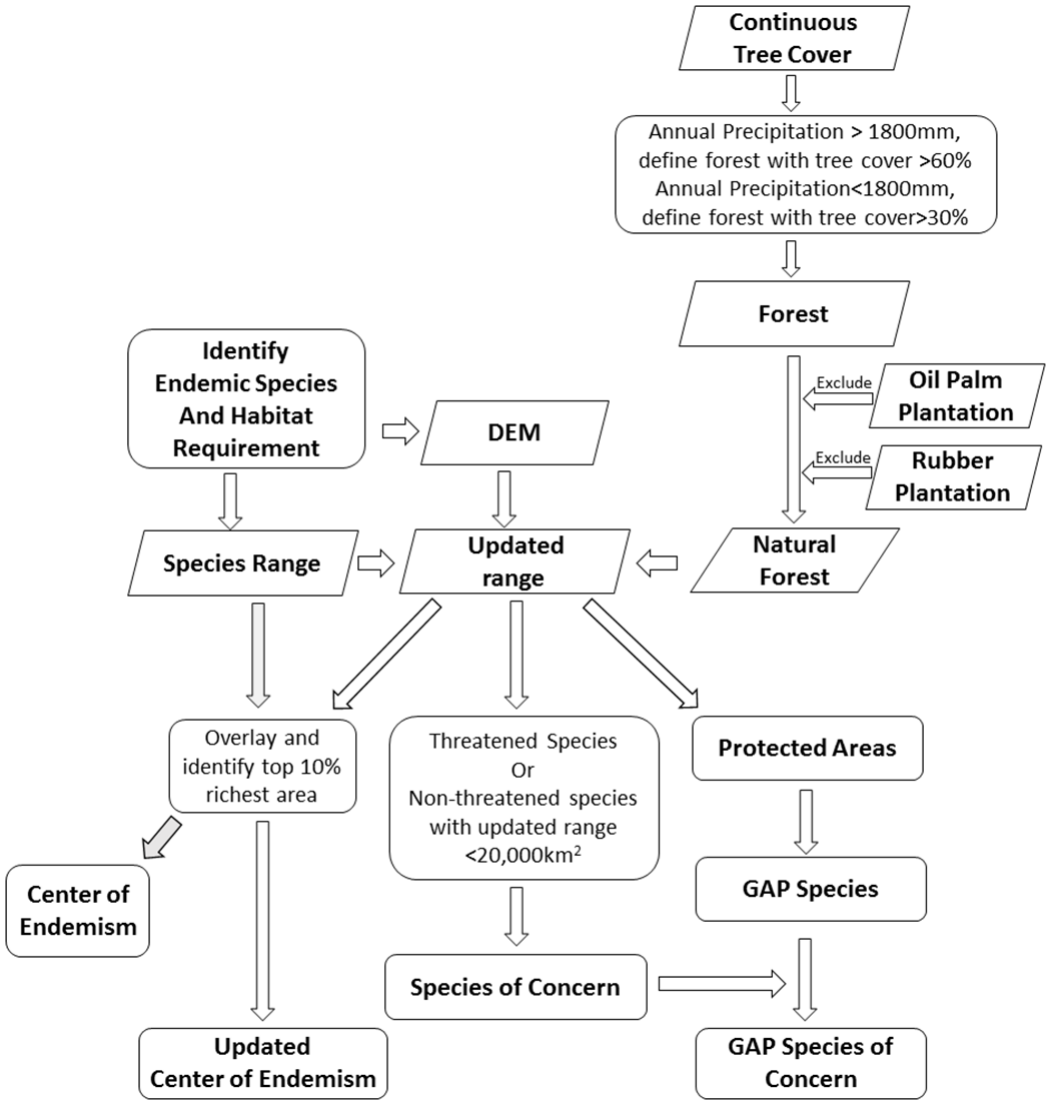
Flow chart of the analysis performed in this paper.

The minimum tree cover needed to define “natural forest” differs widely and the discrepancies are mainly in regions of relatively sparse tree cover[40]. In our study area, the forests in dry areas tend to be more open because of their different species compositions [41]. Lower tree cover thresholds are applied to identify forests in drier regions. To select forested areas correctly, we complemented our layers by testing our four forest layers with different tree cover thresholds against annual rainfall from WorldClim [42]. For regions with different average annual rainfall, we chose the best performing tree cover thresholds to identify forests (Fig 2).

To assess the resultant map of forest cover, we need to address two kinds of errors. The first are errors of omission—places where we exclude natural forest areas from a species’ range. The other are errors of commission, where we include as forests land covers such as plantations that are likely unsuitable habitats.

#### Errors of omission

To address these, we chose points within protected areas across a 1° latitude-longitude grid across the entire area. We then examined each point using high-resolution Google Earth imagery to ensure that all areas within 90m of the point were continuous, natural forest, using imagery from 2012 onwards. At this scale, human encroachments, including tree plantations, were obvious. If present, we selected other points. The final dataset contained 195 natural forest points.

We considered whether we classified these points correctly at the 60% tree cover threshold. The initial results showed that the classification often failed for points where the rainfall was <1800 mm annually, where forests naturally tend to be more open. For these areas, we used a 30% threshold, which classified the points correctly. Details of this process and results are in the supplementary materials.

#### Errors of commission

Land cover products and forest layers are often unable to differentiate natural forests from tree plantations [29]. Two major tree crops in Southeast Asia are rubber and oil palm [16], followed by relatively small tracts of other types, such as teak, acacia and fruit trees. Although rubber plantations have extended to the north far beyond their optimal range [17], rubber usually occurs in the region where there is >2000mm annual precipitation [43, 44] and most consider 1500mm annual rainfall to be the lower limit for commercial production. We randomly selected the locations of 200 oil palm mills from the Global Forest Watch [45] dataset and manually checked each using high-resolution Google Earth imagery to find nearby oil palm plantations. For rubber, we chose three regions, Yunnan from China, Central Highlands from Vietnam, and Ubon Ratchathani from Thailand. We identified rubber plantations, other than those Li and Fox identified in these regions, using high resolution Google Earth imagery and randomly chose 200 points with a minimum distance of 1km between each. We used these as verification points to estimate the error of commission for our forest layer.

### Comparison with other global forest products

To assess the effect of using alternative forest maps, we looked at the Land Cover 2010 map that built upon ESA-CCI from the European Space Agency (ESA)-Climate Change Initiative (CCI). We extracted all the forest-related categories (50, 60, 61, 62, 70, 160, and 170) to make a forest layer for this product (ESA-CCI forest). ESA-CCI is a more conservative estimate of forests when compared to other global land cover products such as MCD12Q1 and the PALSAR-based forest map [29].

### Refining ranges and patterns of biodiversity

We refined the species range maps by clipping them to the species’ elevational limits and remaining natural forests. We used elevational data from the 90m-resolution NASA Shuttle Radar Topographic Mission (SRTM) downloaded from http://earthexplorer.usgs.gov/ and the forest layers that we updated (see above). We calculated range size after each refining step. We then summed all species ranges for each step to understand the patterns of biodiversity and how these patterns change after range refinement (Fig 2).

In addition to threatened species listed by IUCN, we also consider non-threatened species that have limited ranges (< 20,000 km^2^) after refinement using elevation and habitat to be species of concern. To assess how well these species are protected, we downloaded protected area boundaries from the World Database of Protected Areas [46], excluding those listed as only “proposed.” For each species, we calculated how much of its range intersects with protected areas (Fig 2).

### Difference in conservation priorities after incorporating more information

To identify the differences in conservation priorities between using the original ranges and those incorporating species’ elevational preferences and remaining forests, we selected the most biodiverse areas until our sample contained 10% of the forest area. The selection first added the pixels with the highest number of target species, then those with the next highest until the process reached the 10% limit. We did this for both the original ranges and those after refinement by elevation and by forest. We refer to the selected areas as “endemism centres” (Fig 2).

## Results

### Elevation, forest, and protected areas

Fig 1 shows the region, its political boundaries, and the data layers used in our analyses. There are two maps for forest cover with a third comparing the two. Supplementary materials (A in S1 File) detail how we calibrated the forest layer and the accuracy matrix. The major differences between our forest map and the ESA-CCI forest map are in the dry areas with annual precipitation lower than 1800mm (Fig A in S1 File). These areas in eastern Yunnan and northern Vietnam and Laos are the same areas where Sexton et al. [40] find considerable disagreement in various global maps of forest cover.

### Refining ranges

**Fig 3:**
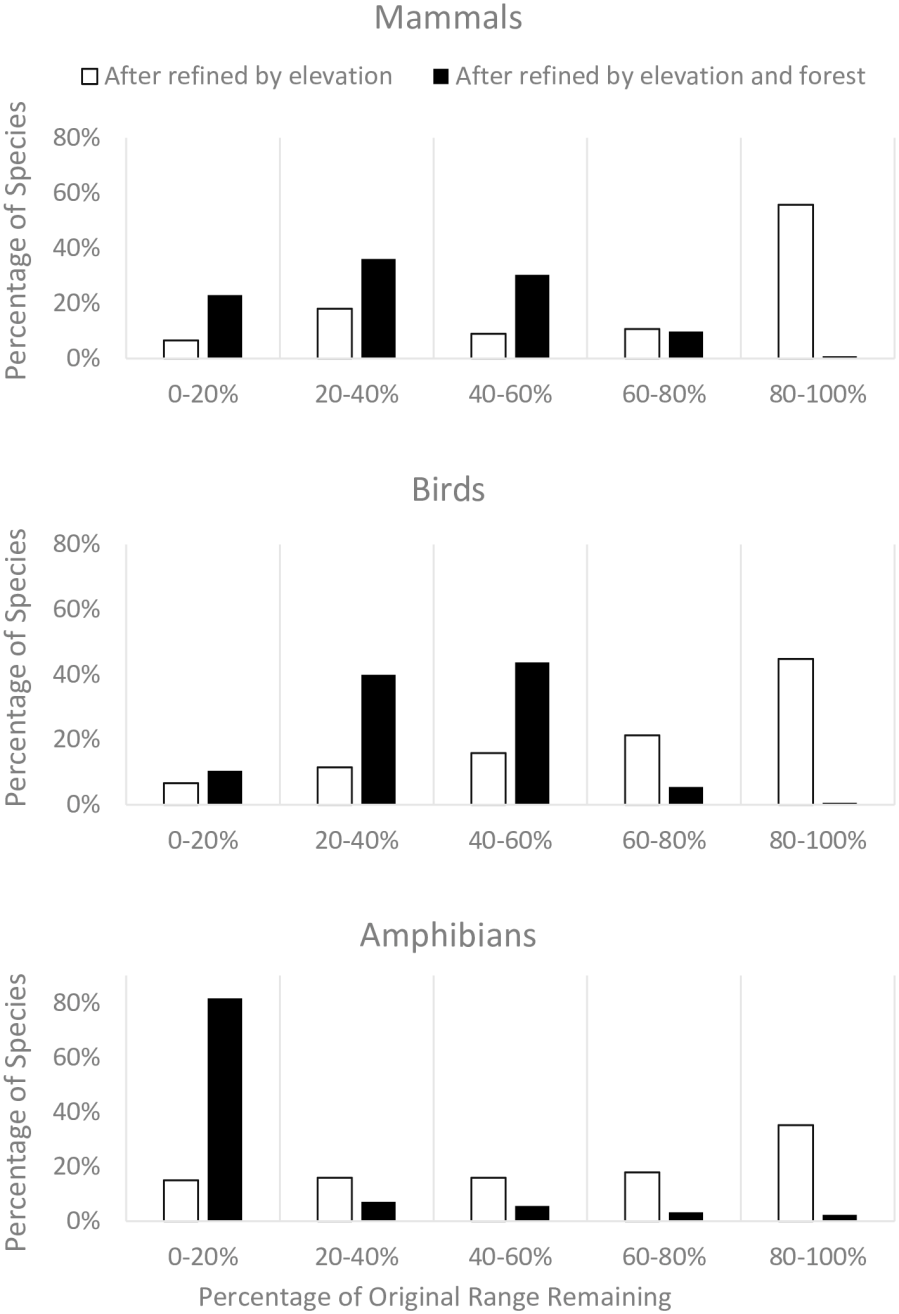
Percentage of species with different levels of remaining ranges after each step of refining.

Fig 3 shows the fraction of original ranges after accounting for elevational range and remaining natural forests. The average refined ranges are 13%, 36%, and 39% of the original ranges for amphibians, mammals, and birds respectively. The majority of the species still had more than 80% of their range within their elevational preference (Fig 3). The major reduction in range size happened during the refinement by forest cover. Supplementary materials tabulate the changes for each species.

**Fig 4:**
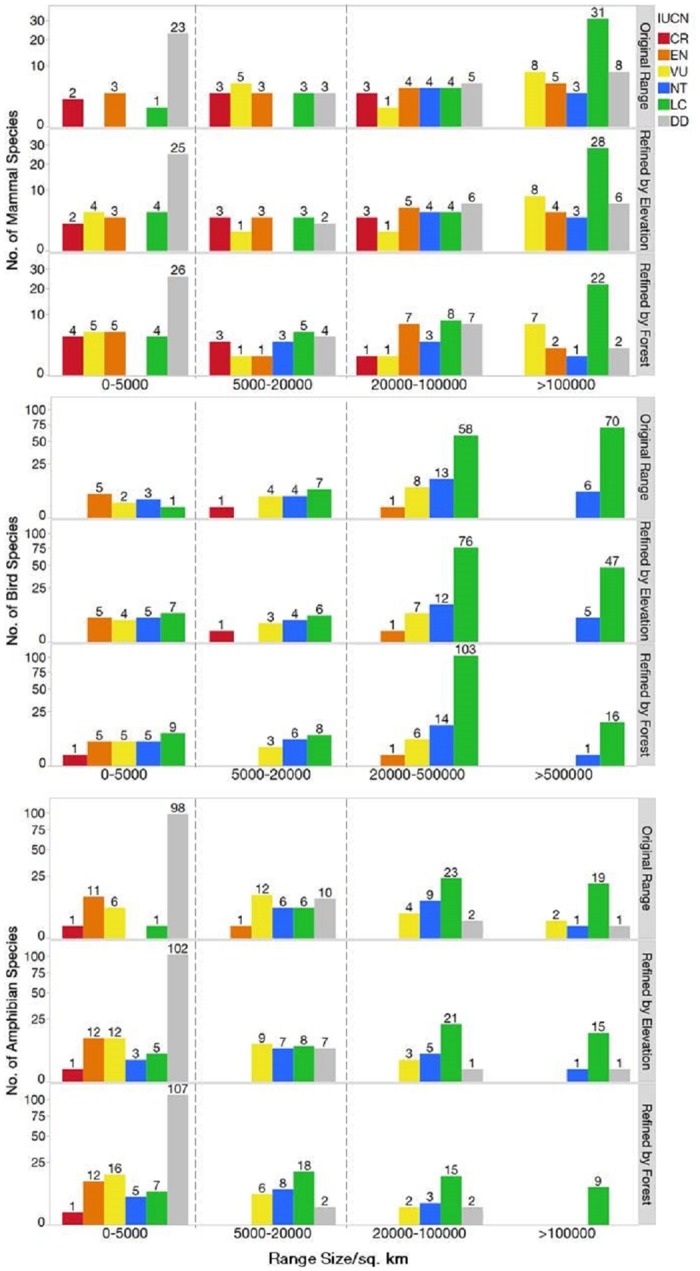
Number of species in each IUCN category, with their original ranges, then after refinement by elevation and then remaining forest. The scale is square root transformed. The dotted lines divide the ranges into three groups, 0-5000km^2^, 5000-20000km^2^, and above. When a species range falls under 20,000km^2^, we treat it as a species of concern.

Fig 4 shows the distributions of original range sizes, then ranges refined by elevation, then by remaining natural forest, and by species' category of threat. As one expects, species with smaller ranges are more likely to be classified into a higher level of threat than those with large ranges. Some species with relatively large ranges have high levels of threat and these include large-bodied birds and mammals, which often exist at naturally low population densities and are heavily hunted. There are large numbers of data deficient species, especially of amphibians, which have small geographical ranges. While it would be wrong to assume that all data deficient species are classified as such because they have small ranges—and thus are likely to be rare within the small ranges [2]—clearly that is a strong possibility.

Range sizes inevitably shrink when refined by elevation and by remaining forest. The IUCN criteria includes range size, but does not refined range size directly using high precision variables. They may well do so indirectly, via the expert opinions of those who assess the species. Nonetheless, many species move into range size classes where the majority of the comparable species have a higher level of threat than IUCN attributes to them. For mammals, there are respectively two (*Biswamoyopterus biswasi*, *Rhinopithecus strykeri*), three (*Hadromys humei*, *Hapalomys longicaudatus*, *Laonastes aenigmamus*), and one species (*Arielulus aureocollaris*) of critically endangered, endangered, and least concerned species with ranges <5,000 km^2^. When refined by elevation and remaining forest, the numbers increase to two additional critically endangered (*Trachypithecus delacouri*, *Rhinopithecus avunculus*), two more endangered (*Hipposideros halophyllus*, *Trachypithecus shortridgei*), five more vulnerable (*Craseonycteris thonglongyai*, *Trachypithecus laotum*, *Niviventer cameroni*, *Hipposideros khaokhouayensis*, *Arielulus societatis*), and three more least concern (*Dremomys gularis*, *Anourosorex assamensis*, *Leopoldamys milleti*).

Overall, after accounting for elevation and remaining forest, 61 mammals, 42 birds, and 182 amphibians have remaining ranges <20,000 km^2^. Most of these species have their remaining range <5,000 km^2^ (Fig 3; Table 1). We created a final list for species of concern that included the threatened species identified by IUCN (37 mammal, 21 bird, and 37 amphibians) and additionally the non-threatened species (as well as data deficient species) with a refined range <20,000 km^2^ (42 mammal, 28 bird, and 147 amphibians). More species currently deemed non-threatened (46 species plus 7 data deficient species), than threatened species (9 species), have their ranges shifted from above to under 20,000km^2^.

**Table 1:** Number of species in each taxon in different categories and coverage by protected areas.

	Mammal	Bird	Amphibian
**Endemic Species**	122	183	213
**Threatened**	37	21	37
**Data Deficient**	39	0	111
**Refined Range <20,000km**^**2**^	61	42	182
**Refined Range <5,000km**^**2**^	44	24	144
**Species of Concern**	79	49	184
**Average % protection**	24±22	20±14	32±34
**No Protection**	14	2	39
**Data Deficient with no protection**	10	0	34

Our species of concern include 79 mammals, 49 birds, and 184 amphibians. More than 50% of them IUCN currently designates as non-threatened. Among all the non-threatened species identified by IUCN, 84% of amphibians, 49% of mammals, and 17% of birds are considered species of concern. This increases the current IUCN estimates significantly. Especially for data deficient species, 93% of them have their ranges <20,000km^2^, and 89% are <5,000km^2^.

### Protection for different taxa

Fig 1C shows the distribution of protected areas in Southeast Asia, and Table 2 shows how well different groups are protected. (Tables B-D in S1 File tabulate the details for each species.) Some 22% of mammals, 14% of birds, and 35% of amphibians have less than 10% of their refined ranges protected. Only two bird species (*Pitta gurneyi*, *Spelaeornis longicaudatus*) have no part of their ranges within protected areas, while 14 mammals and 39 amphibians do. Only six mammals, four birds, and six amphibians have more than 80% of their ranges protected.

**Table 2:** Numbers of species that have <10% or >80% of their refined ranges protected.

PA Coverage	Species of Concern				Other		
	Threatened	Non-Threatened <20,000 km^2^	Data Deficient <20,000 km^2^	Total	Non-Threatened> 20,000km^2^	Data Deficient> 20,000km^2^	Total
Mammals							
<10%	6	2	15	23	3	1	4
>80%	1	1	4	6	0	0	0
Total	37	12	30	79	34	9	43
Birds							
<10%	7	7		14	12		12
>80%	1	3		4	0		0
Total	21	28		49	134		134
Amphibians							
<10%	14	11	47	72	2	0	2
>80%	3	3	29	35	0	0	0
Total	37	38	109	184	27	2	29

Table 2 also shows how protected areas cover different subsets of each taxon. There is no significant difference in protection rates between threatened and non-threatened species no matter the size of the remaining ranges (t-test, *p*>0.05). For species that have refined ranges < 20,000 km^2^, two features are of note. First, consider the fractions of species with protected range <10% between what IUCN deems non-threatened with those threatened: 2 of 12 versus 6 of 37 mammals, 7 of 28 versus 7 of 21 birds, and 11 of 38 versus 14 of 38 amphibians. In each case, the fractions are very similar—a pattern confirmed when one calculates the average fractions of ranges protected (t-test, *p*>0.05). Simply, putatively non-threatened species do not have more of their ranges protected that do comparable species that are threatened. The existing protected area network fails to emphasize the coverage of either threatened species listed by IUCN or the non-threatened species with higher threat because of their limited remaining ranges.

Second, consider the fractions of data deficient species. For both amphibians and mammals, about half the data deficient species have <10% of their estimated ranges protected. There are no data deficient birds.

### Patterns of biodiversity and conservation priority

**Fig 5:**
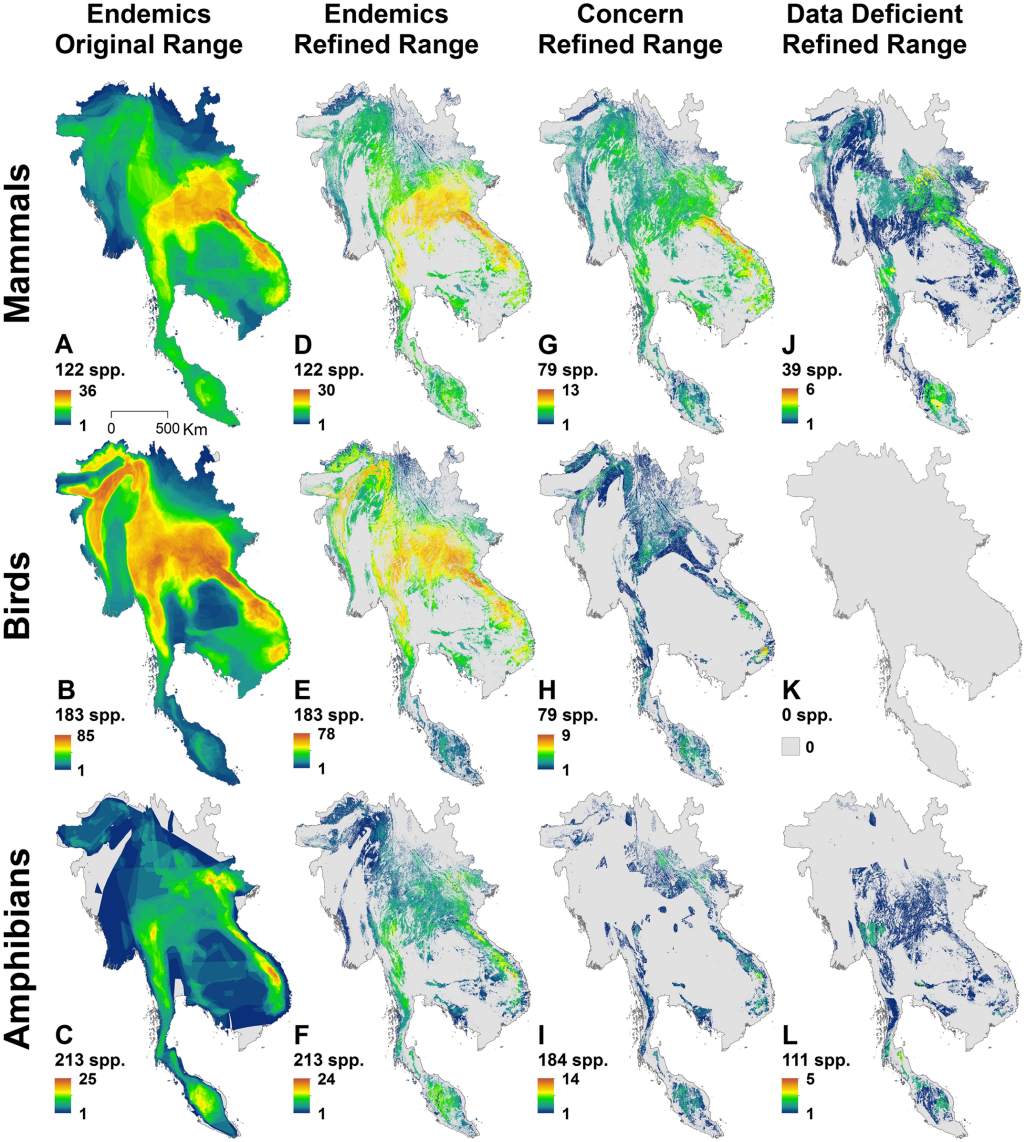
Patterns of biodiversity in Southeast Asia. A-C, using the original range maps from IUCN or BirdLife International for all the endemic species. D-F, using the range maps after refining by elevational range and forest for each species. G-I, using the refined range maps for the species of concern (threatened species and non-threatened species with range<20,000km^2^). J-L, using the refined range maps for data deficient Species.

The mountainous areas of Southeast Asia have high numbers of endemic mammals, birds, and amphibians (Fig 5). The Hoang Lien Son Range in northern Vietnam and the Annamite Range, the principal mountain range of Indochina, and the boundary between Laos and Vietnam are the richest areas for all three taxa. Beyond these mountain ranges, endemic mammals also concentrate in the northern regions of Laos, Vietnam, and Thailand. They extend south to Malay Peninsula through Bilauktaung Range and have another concentration in the Da Lat Plateau. Birds are similar to mammals, but extend further south and north. They have a concentration in the mountain ranges of the Arakan Yoma, the Chin Hills, and Mizo and Naga Hills along the border with Myanmar and India. This is the Mizoram-Manipur-Kachin rainforest ecoregion [47].

The pattern for amphibians is substantially different from birds and mammals. The highest richness areas are relatively concentrated and isolated from each other. The south and central Annamite Range between Ban Vangchang in Laos and Thanh Lang Xa in Vietnam have the most endemic amphibians, followed by the Central Malay Peninsula. Many regions have not been well-surveyed for amphibians. High endemism may also exist in less well explored and surveyed areas.

### Species of concern

The species of concern concentrate in different places (Fig 5G–5I) than for the endemic species overall (Fig 5D–5F). Species of concern for the three taxa all have their highest concentration along the Annamite Range. Mammals have the highest number of species of concern in the north of this range while birds and amphibians have the highest richness in the southern tip of this mountain range and Da Lat Plateau.

#### Data deficient species

About 32% of mammals and 52% of amphibians (Table 1) are data deficient. Such mammals mainly concentrate near the border between China, Vietnam and Laos, northern Annamite Range, Malay Peninsula, and southern Myanmar (Fig 5J). Data deficient amphibians mainly are in the Malay Peninsula, the southern tip of Thailand, and southern Myanmar (Fig 5L). It is possible that many undescribed species exist in un-surveyed parts of the region.

#### Difference in conservation priorities after incorporating more information

**Fig 6:**
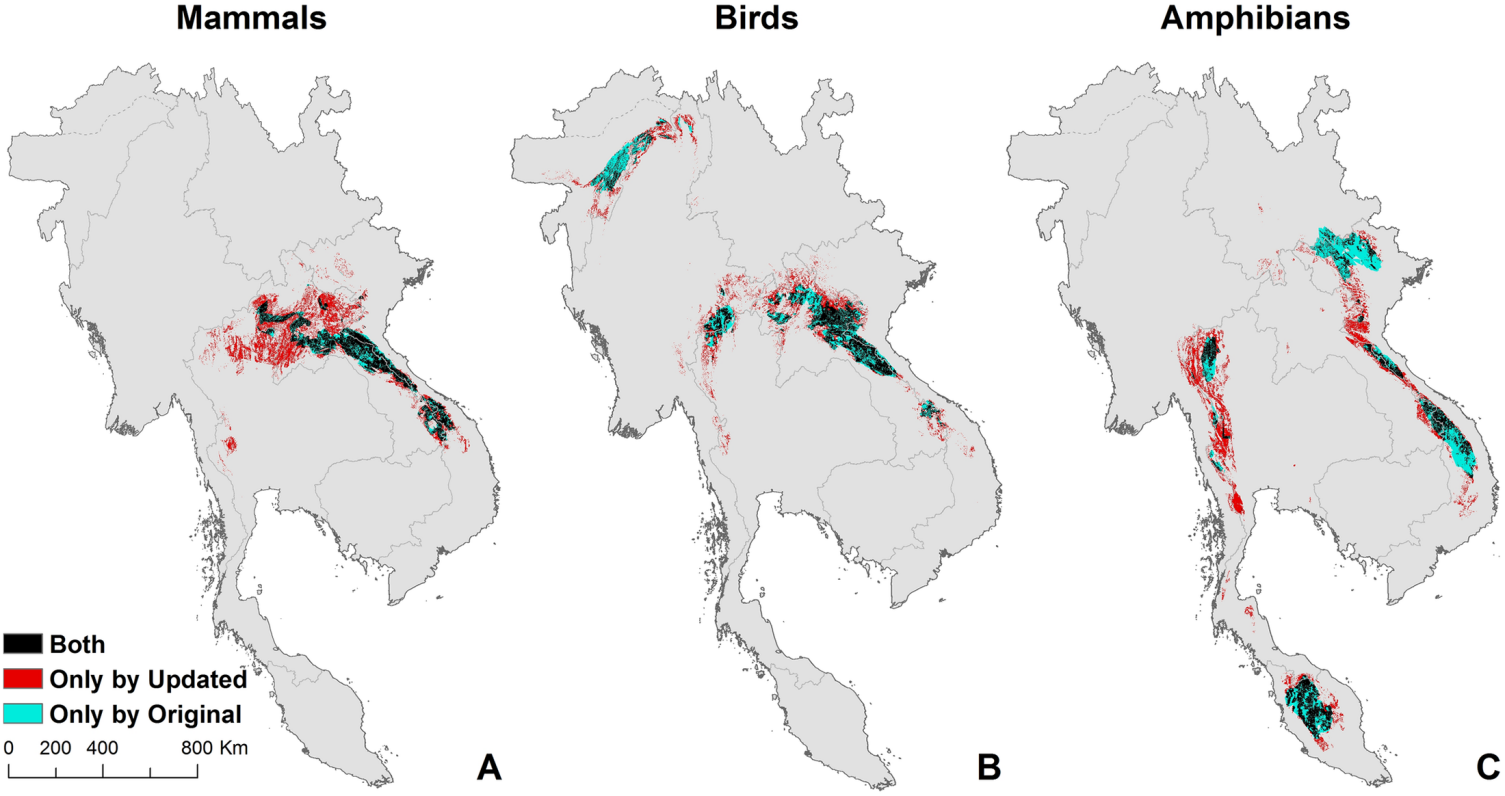
Comparisons of endemism centres using different range maps. The two endemism centres for each taxon were identified using the original ranges and updated ranges respectively. Black areas are agreed upon by both methods. Red areas are the endemism centre identified only by overlaying the updated ranges and blue areas are for original ranges only.

We calculated endemism centres from the original ranges (Fig 5A–5C) and after refinement by elevation and forest (Fig 5D–5F). Fig 6 shows the areas in agreement or otherwise for the three taxa. Green and red areas are the endemic identified using updated range maps. All three taxa have part of the endemic centre in the Annamite Range of Laos and Cambodia. Mammals extend more to north Thailand while birds also have another high concentration on the border between India and Myanmar. The Dawna range of eastern Myanmar and adjacent Thailand and the Malaysia peninsular are only identified as endemism centres for amphibians.

There are broad similarities between the two approaches and among the three maps. Simply using the original IUCN ranges overlooks many regions of importance, which are shown in red. For mammals, this mainly concentrates in north Thailand, Laos and Vietnam, as well as southern Thailand. For birds, the overlooked areas are in Dawna range in western Thailand and southern tip of Annamite Range. For amphibians, they are in northern and southern tips of Annamite Range, as well as the Dawna range in Thailand. The blue areas were considered unsuitable for species because they were either outside the elevational range or devoid of natural forests. The total area of the centres of endemism that is protected is 11326 km^2^. Protected areas cover 2982 km^2^, 4606 km^2^, 6036 km^2^ of the priority areas of mammals, birds, and amphibians respectively.

## Discussion

The expert opinion system that the IUCN Red List employs factors in specific knowledge of diverse threats, which could involve whether the species is hunted, how specific are its habitat requirements, how much habitat remains within its broad range, and whether habitat is being lost or numbers are decreasing for other reasons. IUCN uses an original range of 20,000km^2^ as an important benchmark for endangerment. The majority of threatened species have ranges smaller than this and are in places where there is continuing loss of habitat.

Small range size alone is not sufficient for a “threatened” listing. Nor is it necessary. For example, IUCN classifies some birds and mammals as critically endangered when their original ranges are >20,000km^2^ and some as vulnerable when their original ranges are >100,000 km^2^. These species are often large-bodied ones that are hunted or persecuted in other ways. For example: the vulture, *Gyps bengalensis*, is critically endangered, had a range of > 4,000,000 km2—and was common within it until recently, when poisoning massively depleted its numbers[48]. Remote sensing cannot assess such threats.

Nevertheless, many species are endangered due to habitat loss and degradation. This is simpler to quantify then hunting pressure, though that can be a significant threat to many taxa even within intact forest regions. Thus, the lack of explicit information on elevational range, habitat remaining within the range of a species, how fragmented is that range, and how much of that range is protected, is a significant limitation that remote sensing can help overcome.

Our threshold for designating species of concern is also 20,000km^2^. We understand that the criteria for endangerment that IUCN employs are based on the original ranges, not our refined ones, which will inevitably be smaller. Our use of the same extent is not to confuse it, but merely to identify species that IUCN does not deem threatened and yet which have small enough geographical ranges that they might qualify, or be classified at a higher risk than at present.

There are substantial numbers of species (Fig 4) that, once their range is refined, warrant a re-examination of their IUCN listing. Indeed, there are four mammals, nine birds, and seven amphibians that IUCN deems to be species of least concern yet have refined ranges <5,000km^2^ (mammals: *Dremomys gularis*, *Anourosorex assamensis*, *Leopoldamys milleti*, *Arielulus aureocollaris*; birds: *Alcippe danisi*, *Alcippe klossi*, *Arborophila campbelli*, *Arborophila cambodiana*, *Carduelis monguilloti*, *Garrulax annamensis*, *Garrulax peninsulae*, *Psilopogon chersonesus*, *Spelaeornis oatesi*; amphibians: *Amolops archotaphus*, *Amolops mengyangensis*, *Hylarana milleti*, *Kurixalus bisacculus*, *Leptolalax melanolecus*, *Microhyla marmorata*, *Theloderma andersoni*).

Our results are double-edged. First, the actual ranges are small, indeed small enough that IUCN classifies most other species with comparable, refined range sizes as threatened. Second, as Fig 3 shows, only a small part (13–39% on average) of their original range still has potential habitat. Likely, human actions have destroyed the remainder. Habitat is likely to be continuing to be lost—something that remote sensing can best confirm.

Some may assert that decisions on the risks experienced by individual species may be correct even if the assessors did not use geographical data explicitly. Even were this assertion correct, we would argue that it is better to use explicit standardised quantitative criteria whenever possible rather than rely on indirect judgements, which may be less precise and more vulnerable to bias. Transparency in the process is important. In any case, we doubt the assertion for three reasons.

First, refining ranges by elevation limits and remaining habitat cover greatly alters the estimates of remaining range. After accounting for suitable habitats, most species (60% of mammals, 51% of birds, and 60% of amphibians) have their refined ranges <40% of those originally published (Fig 3). Most of the range reduction happened during the refinement by remaining forests rather than by elevational ranges (Fig 3). Thus, the difference between the range as IUCN reports it and the remaining range mainly relies on the knowledge of where forest remains. We recognise, of course, that even these estimates of remaining habitat may be too optimistic: a species may have particular habitats that further restrict its range.

Second, our results show that refining ranges by elevation and habitat *differentially* alters how much range remains. Some species have proportionately considerably smaller ranges than do others following the same process. Consequently, they likely have a considerably greater risk of extinction than hitherto appreciated.

Third, one might answer that IUCN is making consistent decisions by arguing that those species that it does not consider threatened, but that have small refined ranges, might be disproportionately protected by the network of protected areas. Table 2 rejects that possibility.

Finally, data deficient is not a category to ignore, but one that requires extra attention in conservation planning. Many such species have small known ranges and are overlooked by the protected areas. These may be at greater risk than the officially threatened species designated by IUCN, especially so since their data deficient status already suggests that these species are likely to be exceedingly rare.

In sum, the most parsimonious interpretation of our results it that more species are at risk than initially thought. These species of concern need immediate assessment and involve more than half of the mammal and bird species, and about 74% amphibian species that are currently not considered threatened (Table 1).

There are caveats. The critical step in refining species’ ranges comes from estimating how much natural forest cover remains. Across large areas, this poses no challenge—there is no tree cover of any kind. There are classes of problems where determining forest cover is difficult—tree plantations and sparse forest.

The classification of tree plantations as forests is still a problem for large regions, especially at fine geographical scales. To be conservative, we used a relatively high tree cover threshold both for dry and wet regions, and we additionally excluded tree plantations based on other studies. Our map, however, may still miss some of these areas and some recent expansions of plantations. At the same time, we may miss natural forests that have low tree cover, such as some woodlands and savannahs, especially in dry areas. The conservation community would benefit from further improvements to future forest maps, including the differentiating of natural forest and plantations.

### Knowledge from remote sensing modifies conservation priorities

Our results show a substantial difference between the locations of centres of endemism before and after using the remote sensing data (Fig 5). About 20% of the area identified using original mammal ranges, 25% for birds, and 40% for amphibians does not appear to have natural forests (Fig 6). Remote sensing data can clearly reveal human-modified landscapes. Given the limited conservation resources, incorporating such data for a more accurate delineation of priorities can produce more practical and efficient conservation plans, especially at local scales.

Compared to the lowland forest in Southeast Asia, which is among the most diverse biomes in the world, montane forests in Southeast Asia receive considerably less attention [12]. Our study shows that the mountainous areas harbour the highest biodiversity that is restricted to this region (Fig 5). These montane forests are experiencing rapid deforestation [12, 49]. Plantations of rubber are expanding to higher altitudes and drier and colder areas [17] due to China’s success in researching the growth of rubber in non-traditional environments and to increasing global demand. These expansions are a great concern for the endemic species whose survival depends on the integrity of natural forests. Because many countries in the study area use mountain ranges to delineate the country borders, it will require collaboration between countries to reduce threats such as poaching, illegal logging, and transformation of natural forests to plantations. Thus, trans-boundary protected areas are essential where the richest biodiversity straddles country borders.

Habitat loss remains the most serious threat to species in Southeast Asia, to which poaching, invasive species, and climate change add further risks [16]. Rapid land cover changes require quick and repeatable assessments to respond to these threats. Using publicly available datasets for our analysis, our results show two crucial aspects to consider in future priority-setting, 1) species at risk according to the size of their remaining habitats and coverage by protected areas, and 2) areas with high irreplaceability and vulnerability. This quick and simple method can be widely used and replicated. It is especially useful for conservation practitioners with little access to academic resources or budgets to purchase data. With the increase in remote sensing information freely available to the public and through sharing platforms [40, 50] like Global Forest Watch, it empowers practitioners to track the changes frequently and independently. Our methods are not a panacea to solve conservation problems but do provide an efficient way to track the changes in a species’ status that accounts for its habitat needs, land cover changes, and protected area settings.

Although basal species maps produced by the IUCN may have issues, and are produced by “expert assessment” rather than explicit empirical data, our approach improves the accuracy by incorporating relevant environmental data to help refine suitable areas. Whilst this approach is dependent upon the reliability of basal species range maps—which we do not assess here—our approach represents a considerable advance in increasing the accuracy and better representing the distributions of these species. Furthermore, through the development of an ArcToolbox it is easy to standardise the approach and allow replication in other regions, or even the possibility to become integrated into the existing range mapping process.

### A modest, practical proposal

We argue that individual species assessments may be inconsistent and that the conservation priorities based on them are in less than optimal places. Whatever the pros and cons of the arguments we have presented, it would behove those who evaluate species to present estimates of (1) how much of a species range is within its known elevational range, (2) for forest species, how much of that range still has forest cover, and (3) how much of the range is within protected areas. Such data would permit quantitative insights into a species’ risk of extinction, insights that are currently absent.

## Supporting Information

S1 File **A) Mapping Forest and Evaluation. Table A) Confusion matrix and error estimate. Fig A. Validation of forest cover. Tables B-D. Species Information.** The tables list the IUCN status, elevational range, original range, range after refined by elevational range, and remaining forest, coverage from the protected areas (PA), and whether it is considered as a species of concern in our paper.
(DOCX)
