# Supplementary material for: Remotely Sensed Data Informs Red List Evaluations and Conservation Priorities in Southeast Asia
Source: PLoS One. 2016 Aug 3;11(8):e0160566. doi: 10.1371/journal.pone.0160566 (PMC4972393; doi:10.1371/journal.pone.0160566)
Supplement: S1 File — A) Mapping Forest and Evaluation. Table A) Confusion matrix and error estimate. Fig A. Validation of forest cover. Tables B-D. Species Information. The tables list the IUCN status, elevational range, original range, range after refined by elevational range, and remaining forest, coverage from the protected areas (PA), and whether it is considered as a species of concern in our paper. (DOCX) [file pone.0160566.s001.docx]

**A. Mapping Forest and Evaluation**

We identified 1,036,378 km^2^ of forested area, or 39.1% of the study area. The accuracy assessment for the forest map is below.

**Accuracy assessment**

Fig A (A) shows the fraction of validation sites in natural forests correctly classified for 30, 40, 50, and 60% tree cover thresholds against annual rainfall. For example, at 1400mm of rain, the 60% threshold correctly classified only 65% of the validation points whereas the 30% threshold classified 93%. Above 1800mm, all four thresholds nearly always classified the selected points as forests. This dropped rapidly in drier areas. We added drier validation sites until the fractions of dry and wet sites were approximately proportional to their relative areas. Fig A (B) shows the points correctly (solid points) and incorrectly (red points) classified with the 60% threshold against a background where wet areas have >1800mm annual rainfall (blue) and dry areas have <1800mm. Below 1800 mm, the 30% threshold classifies at least 80% of the points correctly. Fig A (C) shows the correct and incorrectly classified points where we employ the 60% threshold for areas with >1800mm of rain and the 30% threshold below this.

Table A summarizes our accuracy assessment. For natural forests, we correctly classified 88.7%, while the other 11.3% were mainly in dry areas with less than 30% tree cover. While we can further lower the tree cover threshold to reduce the omission error, we risk having a larger commission error. Commission errors overestimate a species’ range by including rubber, oil palm, and other likely unsuitable habitats within our estimates of natural forest. We successfully excluded 95% of oil palms and 82% of rubber from our forest classification.

**Comparison with ESA forest map and differences in the following analysis**

We compared different forest cover maps, our own and by ESA-CCI, and found that the discrepancies mainly concentrate in the northern part of the study area, especially in drier areas (Fig 1). ESA-CCI has higher forest cover in Yunnan and northern Cambodia while our map has more forests in northern Laos, Vietnam and the western coast of Myanmar. Our forest map tends to show less forest in dry areas where the tree cover is sometimes lower than 30%. Identification of forests in this kind of environment is extremely hard as the trees are sparse and definitions of forest may change according to which threshold of tree cover to use. The northern study area is among one of the areas with the greatest discrepancies and uncertainties between different forest products.

Use of the ESA-CCI forest layer would affect the three taxa to varying extents. Birds are less impacted than mammals and amphibians. After refined by the ESA-CCI forest layer, 63% of amphibians, 71% of mammals, and 80% of birds have a range size within 20% of the remaining range refined by our forest layer. For birds, the differences range from 41% to 192% when using the two forest maps, while for mammals, the range extends from 31% to 508%, and for amphibians from 10% to 17.9 times the range refined by our forest cover estimates. In most cases, the ESA-CCI overestimates the areas suitable for a species. While this process does not change the conservation status of any mammals or birds as to whether they are classified as species of concern, it changes the status of some amphibians. If we used ESA-CCI, it would upgrade one amphibian (*Hylarana maosonensis*) to a species of concern while downgrading four species (*Leptolalax heteropus*, *Limnonectes poilani*, *Leptobrachium huashen*, *Xenophrys jingdongensis*) to a non-concerned species.

**Table A Confusion matrix and error estimate.**

|  | **Validation Points** | | | | |  |
| --- | --- | --- | --- | --- | --- | --- |
| **Classification** | Natural forest | Rubber | Oil Palm | Total | **Error of Commission** | |
| Natural Forest | 173 | 38 | 10 | 221 | 21.7% | |
| Non-Forest | 22 | 168 | 190 | 380 | 5.8% | |
| Total | 195 | 206 | 200 | 601 |  | |
| **Error of Omission** | 11.3% | 18.4% | 5.0% |  |  | |
| **Overall Accuracy** |  |  |  |  | 88.4% | |


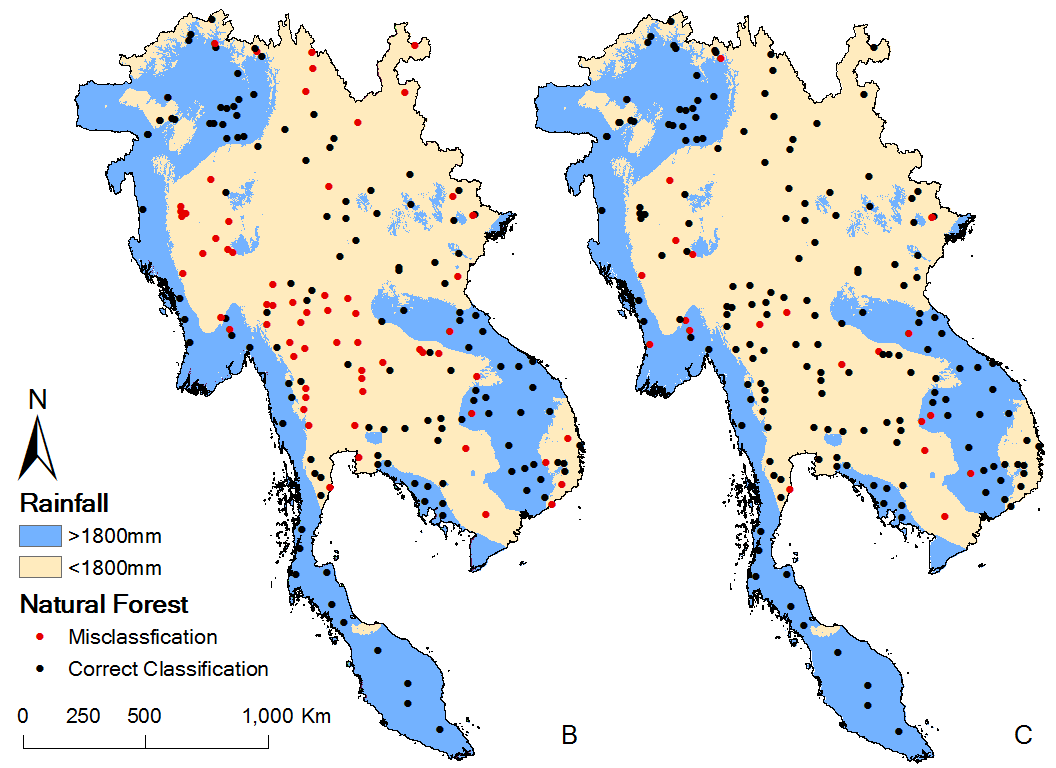


**Fig A. Validation of forest cover.** (A) Fraction of correctly classified forest points versus mean annual rainfall. (B) Correctly (black point) and incorrectly (red point) classified points using a 60% forest cover threshold. (C) The same, using the 60% threshold for areas with >1800 mm of rain and 30% for areas with <1800mm. Blue areas have >1800 of mean annual rainfall while beige areas have <1800 mm.

A

**Table B. Mammal Information**

| **Species** | **Family** | **IUCN** | **Elevational Range** | | **Original Range** | **Refined by Elevation** | **Refined by Forest** | **PA** | **PA**  **Coverage** | **Species of**  **Concern** |
| --- | --- | --- | --- | --- | --- | --- | --- | --- | --- | --- |
|  |  |  | **Min/m** | **Max/m** | **km^2^** | **km^2^** | **km^2^** | **km^2^** |  |  |
| *Anourosorex assamensis* | SORICIDAE | LC | 1500 | 3100 | 17604 | 3008 | 1109 | 0 | 0% | 1 |
| *Arielulus aureocollaris* | VESPERTILIONIDAE | LC | 1200 | 2000 | 2503 | 407 | 389 | 362 | 93% | 1 |
| *Arielulus societatis* | VESPERTILIONIDAE | VU | 16 | 516 | 12278 | 4141 | 2804 | 299 | 11% | 1 |
| *Berylmys berdmorei* | MURIDAE | LC | 200 | 2000 | 660032 | 447602 | 271414 | 79845 | 29% | 0 |
| *Berylmys mackenziei* | MURIDAE | DD | 1200 | 3000 | 99967 | 26526 | 16187 | 1108 | 7% | 1 |
| *Biswamoyopterus biswasi* | SCIURIDAE | CR | 100 | 350 | 3761 | 492 | 155 | 0 | 0% | 1 |
| *Blarinella wardi* | SORICIDAE | LC | 1600 | 3000 | 114883 | 72206 | 35509 | 2373 | 7% | 0 |
| *Callosciurus pygerythrus* | SCIURIDAE | LC | 500 | 1650 | 535122 | 173492 | 96323 | 11299 | 12% | 0 |
| *Callosciurus quinquestriatus* | SCIURIDAE | NT | 1000 | 9999 | 40862 | 36137 | 24703 | 1556 | 6% | 0 |
| *Cannomys badius* | SPALACIDAE | LC | 0 | 4000 | 1015355 | 1014846 | 460327 | 81489 | 18% | 0 |
| *Chimarrogale hantu* | SORICIDAE | NT | 200 | 9999 | 24713 | 20150 | 17334 | 2326 | 13% | 1 |
| *Chodsigoa caovansunga* | SORICIDAE | DD | 1300 | 2000 | 980 | 228 | 194 | 0 | 0% | 1 |
| *Chodsigoa parva* | SORICIDAE | DD | 2750 | 3250 | 71 | 22 | 9 | 0 | 0% | 1 |
| *Chrotogale owstoni* | VIVERRIDAE | VU | 0 | 9999 | 219296 | 219282 | 130137 | 18184 | 14% | 1 |
| *Craseonycteris thonglongyai* | CRASEONYCTERIDAE | VU | 0 | 500 | 7954 | 7779 | 182 | 57 | 31% | 1 |
| *Crocidura indochinensis* | SORICIDAE | LC | 1200 | 2400 | 659230 | 150393 | 90941 | 8599 | 9% | 0 |
| *Crocidura malayana* | SORICIDAE | LC | 0 | 9999 | 173680 | 173580 | 67464 | 12237 | 18% | 0 |
| *Crocidura negligens* | SORICIDAE | LC | 0 | 1000 | 195548 | 189981 | 69847 | 16165 | 23% | 0 |
| *Dacnomys millardi* | MURIDAE | DD | 1050 | 3000 | 34315 | 17323 | 11550 | 2430 | 21% | 1 |
| *Dendrogale murina* | TUPAIIDAE | LC | 0 | 1500 | 203480 | 200945 | 72972 | 17604 | 24% | 0 |
| *Dremomys gularis* | SCIURIDAE | LC | 2500 | 3000 | 42688 | 823 | 766 | 460 | 60% | 1 |
| *Eothenomys cachinus* | CRICETIDAE | LC | 2300 | 3200 | 24962 | 7650 | 6355 | 643 | 10% | 1 |
| *Eudiscopus denticulus* | VESPERTILIONIDAE | DD | 0 | 1000 | 1659 | 1632 | 1001 | 575 | 57% | 1 |
| *Euroscaptor klossi* | TALPIDAE | LC | 0 | 9999 | 668535 | 668535 | 409692 | 75100 | 18% | 0 |
| *Euroscaptor parvidens* | TALPIDAE | DD | 550 | 1050 | 1392 | 752 | 218 | 16 | 7% | 1 |
| *Hadromys humei* | MURIDAE | EN | 900 | 1300 | 2051 | 572 | 362 | 0 | 0% | 1 |
| *Hadromys yunnanensis* | MURIDAE | DD | 0 | 9999 | 2705 | 2705 | 1375 | 412 | 30% | 1 |
| *Hapalomys longicaudatus* | MURIDAE | EN | 250 | 750 | 2084 | 532 | 275 | 85 | 31% | 1 |
| *Harpiocephalus mordax* | VESPERTILIONIDAE | DD | 0 | 9999 | 162918 | 162910 | 76410 | 16897 | 22% | 0 |
| *Hesperoptenus blanfordi* | VESPERTILIONIDAE | LC | 0 | 1000 | 441128 | 422476 | 165850 | 49046 | 30% | 0 |
| *Hesperoptenus doriae* | VESPERTILIONIDAE | DD | 0 | 1000 | 6073 | 6012 | 2912 | 66 | 2% | 1 |
| *Hipposideros grandis* | HIPPOSIDERIDAE | DD | 0 | 9999 | 185983 | 185983 | 90555 | 7284 | 8% | 0 |
| *Hipposideros halophyllus* | HIPPOSIDERIDAE | EN | 0 | 1000 | 9355 | 9351 | 2635 | 1558 | 59% | 1 |
| *Hipposideros khaokhouayensis* | HIPPOSIDERIDAE | VU | 180 | 400 | 17746 | 4281 | 1315 | 204 | 16% | 1 |
| *Hipposideros nequam* | HIPPOSIDERIDAE | DD | 0 | 9999 | 68 | 68 | 1 | 0 | 0% | 1 |
| *Hipposideros rotalis* | HIPPOSIDERIDAE | LC | 0 | 9999 | 29367 | 29367 | 17150 | 4481 | 26% | 1 |
| *Hipposideros turpis* | HIPPOSIDERIDAE | NT | 0 | 1000 | 48202 | 47723 | 15031 | 1160 | 8% | 1 |
| *Hoolock leuconedys* | HYLOBATIDAE | VU | 0 | 2700 | 281195 | 272628 | 160056 | 16132 | 10% | 1 |
| *Hylobates lar* | HYLOBATIDAE | EN | 0 | 1500 | 562941 | 552530 | 233652 | 66750 | 29% | 1 |
| *Hylobates pileatus* | HYLOBATIDAE | EN | 0 | 1500 | 121517 | 121474 | 41737 | 22170 | 53% | 1 |
| *Hylopetes phayrei* | SCIURIDAE | LC | 0 | 1500 | 1048014 | 1006748 | 498323 | 86861 | 17% | 0 |
| *Kerivoula kachinensis* | VESPERTILIONIDAE | LC | 100 | 800 | 570150 | 319961 | 166571 | 22087 | 13% | 0 |
| *Kerivoula krauensis* | VESPERTILIONIDAE | DD | 0 | 1000 | 734 | 670 | 579 | 497 | 86% | 1 |
| *Kerivoula lenis* | VESPERTILIONIDAE | LC | 0 | 1000 | 115628 | 110598 | 46314 | 6591 | 14% | 0 |
| *Kerivoula titania* | VESPERTILIONIDAE | LC | 0 | 1600 | 562037 | 555403 | 277503 | 49058 | 18% | 0 |
| *Laonastes aenigmamus* | DIATOMYIDAE | EN | 0 | 1000 | 2249 | 2240 | 795 | 795 | 100% | 1 |
| *Leopoldamys milleti* | MURIDAE | LC | 1000 | 9999 | 5654 | 3582 | 2636 | 1124 | 43% | 1 |
| *Leopoldamys neilli* | MURIDAE | DD | 100 | 800 | 2142 | 1406 | 631 | 405 | 64% | 1 |
| *Macaca arctoides* | CERCOPITHECIDAE | VU | 50 | 2700 | 1146298 | 1048290 | 575635 | 113701 | 20% | 1 |
| *Macaca leonina* | CERCOPITHECIDAE | VU | 0 | 2000 | 1444778 | 1407754 | 658433 | 137064 | 21% | 1 |
| *Maxomys inas* | MURIDAE | LC | 900 | 9999 | 12646 | 5522 | 5109 | 512 | 10% | 1 |
| *Megaerops niphanae* | PTEROPODIDAE | LC | 100 | 2100 | 1281978 | 930819 | 442046 | 107775 | 24% | 0 |
| *Melogale personata* | MUSTELIDAE | DD | 30 | 1950 | 1916852 | 1699713 | 763179 | 151592 | 20% | 0 |
| *Miniopterus pusillus* | VESPERTILIONIDAE | LC | 0 | 1200 | 970540 | 930729 | 339128 | 94746 | 28% | 0 |
| *Muntiacus feae* | CERVIDAE | DD | 0 | 9999 | 86510 | 86454 | 54702 | 24053 | 44% | 0 |
| *Muntiacus puhoatensis* | CERVIDAE | DD | 900 | 9999 | 467 | 277 | 225 | 0 | 0% | 1 |
| *Muntiacus putaoensis* | CERVIDAE | DD | 700 | 1220 | 108086 | 31476 | 21355 | 3874 | 18% | 0 |
| *Muntiacus rooseveltorum* | CERVIDAE | DD | 700 | 2000 | 380267 | 227708 | 143239 | 21315 | 15% | 0 |
| *Muntiacus truongsonensis* | CERVIDAE | DD | 1000 | 9999 | 288729 | 112136 | 78993 | 11863 | 15% | 0 |
| *Muntiacus vuquangensis* | CERVIDAE | EN | 0 | 1000 | 110003 | 89048 | 52216 | 13824 | 26% | 1 |
| *Murina cyclotis* | VESPERTILIONIDAE | LC | 250 | 1500 | 1780904 | 806503 | 531363 | 124877 | 24% | 0 |
| *Murina harrisoni* | VESPERTILIONIDAE | DD | 0 | 1000 | 370 | 370 | 217 | 176 | 81% | 1 |
| *Murina tubinaris* | VESPERTILIONIDAE | LC | 1200 | 2650 | 877828 | 155907 | 107681 | 14339 | 13% | 0 |
| *Mus pahari* | MURIDAE | LC | 200 | 2000 | 1348212 | 1071013 | 598277 | 94973 | 16% | 0 |
| *Myotis annamiticus* | VESPERTILIONIDAE | DD | 0 | 1000 | 110 | 110 | 100 | 37 | 37% | 1 |
| *Myotis annectans* | VESPERTILIONIDAE | LC | 850 | 1350 | 235545 | 64851 | 53879 | 12578 | 23% | 0 |
| *Myotis hermani* | VESPERTILIONIDAE | DD | 0 | 500 | 3063 | 1094 | 762 | 48 | 6% | 1 |
| *Myotis oreias* | VESPERTILIONIDAE | DD | 0 | 9999 | 535 | 532 | 29 | 15 | 51% | 1 |
| *Myotis siligorensis* | VESPERTILIONIDAE | LC | 914 | 2770 | 1103626 | 322948 | 214729 | 35750 | 17% | 0 |
| *Nesolagus timminsi* | LEPORIDAE | DD | 0 | 1000 | 24822 | 21139 | 16782 | 3648 | 22% | 1 |
| *Niviventer brahma* | MURIDAE | LC | 2000 | 2800 | 32328 | 7559 | 6231 | 1503 | 24% | 1 |
| *Niviventer cameroni* | MURIDAE | VU | 1520 | 2020 | 9975 | 562 | 514 | 85 | 16% | 1 |
| *Niviventer langbianis* | MURIDAE | LC | 200 | 1600 | 631984 | 542769 | 359000 | 90710 | 25% | 0 |
| *Niviventer tenaster* | MURIDAE | LC | 1300 | 3000 | 232236 | 21172 | 16130 | 2741 | 17% | 1 |
| *Nomascus concolor* | HYLOBATIDAE | CR | 500 | 2700 | 26326 | 25057 | 17399 | 4614 | 27% | 1 |
| *Nomascus gabriellae* | HYLOBATIDAE | EN | 400 | 1650 | 94846 | 48017 | 33676 | 9743 | 29% | 1 |
| *Nomascus leucogenys* | HYLOBATIDAE | CR | 200 | 1650 | 51342 | 45315 | 28806 | 10180 | 35% | 1 |
| *Nomascus siki* | HYLOBATIDAE | EN | 30 | 1800 | 26549 | 26042 | 20255 | 7724 | 38% | 1 |
| *Nycticebus bengalensis* | LORISIDAE | VU | 0 | 2400 | 1968412 | 1946479 | 828324 | 162762 | 20% | 1 |
| *Nycticebus pygmaeus* | LORISIDAE | VU | 0 | 1500 | 461373 | 449989 | 234911 | 39191 | 17% | 1 |
| *Ochotona gaoligongensis* | OCHOTONIDAE | DD | 2750 | 3250 | 6122 | 1303 | 1004 | 422 | 42% | 1 |
| *Ochotona nigritia* | OCHOTONIDAE | DD | 2950 | 3450 | 2239 | 445 | 341 | 102 | 30% | 1 |
| *Paracoelops megalotis* | HIPPOSIDERIDAE | DD | 0 | 9999 | 17 | 17 | 0 | 0 | 0% | 1 |
| *Phoniscus atrox* | VESPERTILIONIDAE | NT | 0 | 400 | 174557 | 147994 | 42931 | 6799 | 16% | 0 |
| *Pipistrellus anthonyi* | VESPERTILIONIDAE | DD | 1884 | 2384 | 47 | 20 | 15 | 0 | 0% | 1 |
| *Pipistrellus cadornae* | VESPERTILIONIDAE | LC | 250 | 750 | 510460 | 190012 | 120579 | 23638 | 20% | 0 |
| *Pipistrellus joffrei* | VESPERTILIONIDAE | DD | 0 | 1000 | 4014 | 2278 | 2001 | 0 | 0% | 1 |
| *Pipistrellus paterculus* | VESPERTILIONIDAE | LC | 0 | 1500 | 774413 | 612337 | 286201 | 41876 | 15% | 0 |
| *Pithecheir parvus* | MURIDAE | DD | 0 | 1200 | 101355 | 98787 | 44004 | 6834 | 16% | 0 |
| *Presbytis femoralis* | CERCOPITHECIDAE | NT | 0 | 1000 | 126296 | 125538 | 46806 | 14176 | 30% | 0 |
| *Pseudoryx nghetinhensis* | BOVIDAE | CR | 0 | 1200 | 13995 | 12359 | 9534 | 2770 | 29% | 1 |
| *Pteropus lylei* | PTEROPODIDAE | VU | 0 | 1000 | 106247 | 102137 | 11779 | 6076 | 52% | 1 |
| *Pygathrix cinerea* | CERCOPITHECIDAE | CR | 0 | 9999 | 24681 | 24681 | 11361 | 1295 | 11% | 1 |
| *Pygathrix nemaeus* | CERCOPITHECIDAE | EN | 0 | 1600 | 91505 | 90863 | 55781 | 18969 | 34% | 1 |
| *Pygathrix nigripes* | CERCOPITHECIDAE | EN | 0 | 9999 | 95061 | 95061 | 31828 | 5620 | 18% | 1 |
| *Rhinolophus coelophyllus* | RHINOLOPHIDAE | LC | 0 | 1000 | 425270 | 400052 | 127146 | 51693 | 41% | 0 |
| *Rhinolophus convexus* | RHINOLOPHIDAE | DD | 1000 | 9999 | 7651 | 1952 | 1649 | 154 | 9% | 1 |
| *Rhinolophus paradoxolophus* | RHINOLOPHIDAE | LC | 0 | 9999 | 395850 | 395799 | 202021 | 38351 | 19% | 0 |
| *Rhinolophus robinsoni* | RHINOLOPHIDAE | NT | 0 | 1000 | 47257 | 43775 | 16770 | 2266 | 14% | 1 |
| *Rhinolophus shameli* | RHINOLOPHIDAE | LC | 0 | 1000 | 766127 | 678474 | 222464 | 61592 | 28% | 0 |
| *Rhinolophus siamensis* | RHINOLOPHIDAE | LC | 0 | 1000 | 309658 | 209778 | 126596 | 19279 | 15% | 0 |
| *Rhinolophus yunanensis* | RHINOLOPHIDAE | LC | 0 | 1000 | 401193 | 263109 | 155447 | 35078 | 23% | 0 |
| *Rhinopithecus avunculus* | CERCOPITHECIDAE | CR | 200 | 1200 | 10210 | 5912 | 4724 | 95 | 2% | 1 |
| *Rhinopithecus strykeri* | CERCOPITHECIDAE | CR | 2745 | 3660 | 203 | 74 | 51 | 0 | 0% | 1 |
| *Saxatilomys paulinae* | MURIDAE | DD | 0 | 1000 | 2249 | 2240 | 795 | 795 | 100% | 1 |
| *Suncus malayanus* | SORICIDAE | DD | 0 | 1000 | 170649 | 165158 | 61808 | 10990 | 18% | 0 |
| *Sus bucculentus* | SUIDAE | DD | 0 | 9999 | 368 | 368 | 45 | 0 | 0% | 1 |
| *Tadarida johorensis* | MOLOSSIDAE | VU | 0 | 500 | 80625 | 71472 | 29227 | 5079 | 17% | 1 |
| *Taphozous theobaldi* | EMBALLONURIDAE | LC | 0 | 1200 | 998406 | 671650 | 157608 | 58972 | 37% | 0 |
| *Tonkinomys daovantieni* | MURIDAE | DD | 0 | 500 | 117 | 114 | 92 | 92 | 100% | 1 |
| *Trachypithecus barbei* | CERCOPITHECIDAE | DD | 0 | 9999 | 21027 | 21017 | 10952 | 7487 | 68% | 1 |
| *Trachypithecus delacouri* | CERCOPITHECIDAE | CR | 0 | 1000 | 6008 | 5945 | 2106 | 202 | 10% | 1 |
| *Trachypithecus germaini* | CERCOPITHECIDAE | EN | 0 | 1000 | 342521 | 325292 | 95437 | 33752 | 35% | 1 |
| *Trachypithecus hatinhensis* | CERCOPITHECIDAE | EN | 0 | 9999 | 18903 | 18903 | 10563 | 2297 | 22% | 1 |
| *Trachypithecus laotum* | CERCOPITHECIDAE | VU | 1000 | 9999 | 5592 | 706 | 493 | 119 | 24% | 1 |
| *Trachypithecus obscurus* | CERCOPITHECIDAE | NT | 0 | 1000 | 257716 | 251624 | 103545 | 23895 | 23% | 0 |
| *Trachypithecus phayrei* | CERCOPITHECIDAE | EN | 0 | 9999 | 1107063 | 1106443 | 466495 | 75369 | 16% | 1 |
| *Trachypithecus shortridgei* | CERCOPITHECIDAE | EN | 200 | 2500 | 10535 | 5853 | 4906 | 390 | 8% | 1 |
| *Tragulus versicolor* | TRAGULIDAE | DD | 0 | 500 | 1480 | 960 | 409 | 0 | 0% | 1 |
| *Tragulus williamsoni* | TRAGULIDAE | DD | 0 | 9999 | 316 | 316 | 119 | 0 | 0% | 1 |
| *Uropsilus investigator* | TALPIDAE | DD | 3600 | 4600 | 3186 | 803 | 83 | 54 | 64% | 1 |
| *Viverra megaspila* | VIVERRIDAE | VU | 0 | 500 | 1408099 | 944190 | 298597 | 64223 | 22% | 1 |

**Table C. Bird Information**

| **Species** | **Family** | **IUCN** | **Elevational Range** | | **Original Range** | **Refined by Elevation** | **Refined by Forest** | **PA** | **PA**  **Coverage** | **Species of**  **Concern** |
| --- | --- | --- | --- | --- | --- | --- | --- | --- | --- | --- |
|  |  |  | **Min/m** | **Max/m** | **km^2^** | **km^2^** | **km^2^** | **km^2^** |  |  |
| *Anourosorex assamensis* | SORICIDAE | LC | 1500 | 3100 | 17604 | 3008 | 1109 | 0 | 0% | 1 |
| *Arielulus aureocollaris* | VESPERTILIONIDAE | LC | 1200 | 2000 | 2503 | 407 | 389 | 362 | 93% | 1 |
| *Arielulus societatis* | VESPERTILIONIDAE | VU | 16 | 516 | 12278 | 4141 | 2804 | 299 | 11% | 1 |
| *Berylmys berdmorei* | MURIDAE | LC | 200 | 2000 | 660032 | 447602 | 271414 | 79845 | 29% | 0 |
| *Berylmys mackenziei* | MURIDAE | DD | 1200 | 3000 | 99967 | 26526 | 16187 | 1108 | 7% | 1 |
| *Biswamoyopterus biswasi* | SCIURIDAE | CR | 100 | 350 | 3761 | 492 | 155 | 0 | 0% | 1 |
| *Blarinella wardi* | SORICIDAE | LC | 1600 | 3000 | 114883 | 72206 | 35509 | 2373 | 7% | 0 |
| *Callosciurus pygerythrus* | SCIURIDAE | LC | 500 | 1650 | 535122 | 173492 | 96323 | 11299 | 12% | 0 |
| *Callosciurus quinquestriatus* | SCIURIDAE | NT | 1000 | 9999 | 40862 | 36137 | 24703 | 1556 | 6% | 0 |
| *Cannomys badius* | SPALACIDAE | LC | 0 | 4000 | 1015355 | 1014846 | 460327 | 81489 | 18% | 0 |
| *Chimarrogale hantu* | SORICIDAE | NT | 200 | 9999 | 24713 | 20150 | 17334 | 2326 | 13% | 1 |
| *Chodsigoa caovansunga* | SORICIDAE | DD | 1300 | 2000 | 980 | 228 | 194 | 0 | 0% | 1 |
| *Chodsigoa parva* | SORICIDAE | DD | 2750 | 3250 | 71 | 22 | 9 | 0 | 0% | 1 |
| *Chrotogale owstoni* | VIVERRIDAE | VU | 0 | 9999 | 219296 | 219282 | 130137 | 18184 | 14% | 1 |
| *Craseonycteris thonglongyai* | CRASEONYCTERIDAE | VU | 0 | 500 | 7954 | 7779 | 182 | 57 | 31% | 1 |
| *Crocidura indochinensis* | SORICIDAE | LC | 1200 | 2400 | 659230 | 150393 | 90941 | 8599 | 9% | 0 |
| *Crocidura malayana* | SORICIDAE | LC | 0 | 9999 | 173680 | 173580 | 67464 | 12237 | 18% | 0 |
| *Crocidura negligens* | SORICIDAE | LC | 0 | 1000 | 195548 | 189981 | 69847 | 16165 | 23% | 0 |
| *Dacnomys millardi* | MURIDAE | DD | 1050 | 3000 | 34315 | 17323 | 11550 | 2430 | 21% | 1 |
| *Dendrogale murina* | TUPAIIDAE | LC | 0 | 1500 | 203480 | 200945 | 72972 | 17604 | 24% | 0 |
| *Dremomys gularis* | SCIURIDAE | LC | 2500 | 3000 | 42688 | 823 | 766 | 460 | 60% | 1 |
| *Eothenomys cachinus* | CRICETIDAE | LC | 2300 | 3200 | 24962 | 7650 | 6355 | 643 | 10% | 1 |
| *Eudiscopus denticulus* | VESPERTILIONIDAE | DD | 0 | 1000 | 1659 | 1632 | 1001 | 575 | 57% | 1 |
| *Euroscaptor klossi* | TALPIDAE | LC | 0 | 9999 | 668535 | 668535 | 409692 | 75100 | 18% | 0 |
| *Euroscaptor parvidens* | TALPIDAE | DD | 550 | 1050 | 1392 | 752 | 218 | 16 | 7% | 1 |
| *Hadromys humei* | MURIDAE | EN | 900 | 1300 | 2051 | 572 | 362 | 0 | 0% | 1 |
| *Hadromys yunnanensis* | MURIDAE | DD | 0 | 9999 | 2705 | 2705 | 1375 | 412 | 30% | 1 |
| *Hapalomys longicaudatus* | MURIDAE | EN | 250 | 750 | 2084 | 532 | 275 | 85 | 31% | 1 |
| *Harpiocephalus mordax* | VESPERTILIONIDAE | DD | 0 | 9999 | 162918 | 162910 | 76410 | 16897 | 22% | 0 |
| *Hesperoptenus blanfordi* | VESPERTILIONIDAE | LC | 0 | 1000 | 441128 | 422476 | 165850 | 49046 | 30% | 0 |
| *Hesperoptenus doriae* | VESPERTILIONIDAE | DD | 0 | 1000 | 6073 | 6012 | 2912 | 66 | 2% | 1 |
| *Hipposideros grandis* | HIPPOSIDERIDAE | DD | 0 | 9999 | 185983 | 185983 | 90555 | 7284 | 8% | 0 |
| *Hipposideros halophyllus* | HIPPOSIDERIDAE | EN | 0 | 1000 | 9355 | 9351 | 2635 | 1558 | 59% | 1 |
| *Hipposideros khaokhouayensis* | HIPPOSIDERIDAE | VU | 180 | 400 | 17746 | 4281 | 1315 | 204 | 16% | 1 |
| *Hipposideros nequam* | HIPPOSIDERIDAE | DD | 0 | 9999 | 68 | 68 | 1 | 0 | 0% | 1 |
| *Hipposideros rotalis* | HIPPOSIDERIDAE | LC | 0 | 9999 | 29367 | 29367 | 17150 | 4481 | 26% | 1 |
| *Hipposideros turpis* | HIPPOSIDERIDAE | NT | 0 | 1000 | 48202 | 47723 | 15031 | 1160 | 8% | 1 |
| *Hoolock leuconedys* | HYLOBATIDAE | VU | 0 | 2700 | 281195 | 272628 | 160056 | 16132 | 10% | 1 |
| *Hylobates lar* | HYLOBATIDAE | EN | 0 | 1500 | 562941 | 552530 | 233652 | 66750 | 29% | 1 |
| *Hylobates pileatus* | HYLOBATIDAE | EN | 0 | 1500 | 121517 | 121474 | 41737 | 22170 | 53% | 1 |
| *Hylopetes phayrei* | SCIURIDAE | LC | 0 | 1500 | 1048014 | 1006748 | 498323 | 86861 | 17% | 0 |
| *Kerivoula kachinensis* | VESPERTILIONIDAE | LC | 100 | 800 | 570150 | 319961 | 166571 | 22087 | 13% | 0 |
| *Kerivoula krauensis* | VESPERTILIONIDAE | DD | 0 | 1000 | 734 | 670 | 579 | 497 | 86% | 1 |
| *Kerivoula lenis* | VESPERTILIONIDAE | LC | 0 | 1000 | 115628 | 110598 | 46314 | 6591 | 14% | 0 |
| *Kerivoula titania* | VESPERTILIONIDAE | LC | 0 | 1600 | 562037 | 555403 | 277503 | 49058 | 18% | 0 |
| *Laonastes aenigmamus* | DIATOMYIDAE | EN | 0 | 1000 | 2249 | 2240 | 795 | 795 | 100% | 1 |
| *Leopoldamys milleti* | MURIDAE | LC | 1000 | 9999 | 5654 | 3582 | 2636 | 1124 | 43% | 1 |
| *Leopoldamys neilli* | MURIDAE | DD | 100 | 800 | 2142 | 1406 | 631 | 405 | 64% | 1 |
| *Macaca arctoides* | CERCOPITHECIDAE | VU | 50 | 2700 | 1146298 | 1048290 | 575635 | 113701 | 20% | 1 |
| *Macaca leonina* | CERCOPITHECIDAE | VU | 0 | 2000 | 1444778 | 1407754 | 658433 | 137064 | 21% | 1 |
| *Maxomys inas* | MURIDAE | LC | 900 | 9999 | 12646 | 5522 | 5109 | 512 | 10% | 1 |
| *Megaerops niphanae* | PTEROPODIDAE | LC | 100 | 2100 | 1281978 | 930819 | 442046 | 107775 | 24% | 0 |
| *Melogale personata* | MUSTELIDAE | DD | 30 | 1950 | 1916852 | 1699713 | 763179 | 151592 | 20% | 0 |
| *Miniopterus pusillus* | VESPERTILIONIDAE | LC | 0 | 1200 | 970540 | 930729 | 339128 | 94746 | 28% | 0 |
| *Muntiacus feae* | CERVIDAE | DD | 0 | 9999 | 86510 | 86454 | 54702 | 24053 | 44% | 0 |
| *Muntiacus puhoatensis* | CERVIDAE | DD | 900 | 9999 | 467 | 277 | 225 | 0 | 0% | 1 |
| *Muntiacus putaoensis* | CERVIDAE | DD | 700 | 1220 | 108086 | 31476 | 21355 | 3874 | 18% | 0 |
| *Muntiacus rooseveltorum* | CERVIDAE | DD | 700 | 2000 | 380267 | 227708 | 143239 | 21315 | 15% | 0 |
| *Muntiacus truongsonensis* | CERVIDAE | DD | 1000 | 9999 | 288729 | 112136 | 78993 | 11863 | 15% | 0 |
| *Muntiacus vuquangensis* | CERVIDAE | EN | 0 | 1000 | 110003 | 89048 | 52216 | 13824 | 26% | 1 |
| *Murina cyclotis* | VESPERTILIONIDAE | LC | 250 | 1500 | 1780904 | 806503 | 531363 | 124877 | 24% | 0 |
| *Murina harrisoni* | VESPERTILIONIDAE | DD | 0 | 1000 | 370 | 370 | 217 | 176 | 81% | 1 |
| *Murina tubinaris* | VESPERTILIONIDAE | LC | 1200 | 2650 | 877828 | 155907 | 107681 | 14339 | 13% | 0 |
| *Mus pahari* | MURIDAE | LC | 200 | 2000 | 1348212 | 1071013 | 598277 | 94973 | 16% | 0 |
| *Myotis annamiticus* | VESPERTILIONIDAE | DD | 0 | 1000 | 110 | 110 | 100 | 37 | 37% | 1 |
| *Myotis annectans* | VESPERTILIONIDAE | LC | 850 | 1350 | 235545 | 64851 | 53879 | 12578 | 23% | 0 |
| *Myotis hermani* | VESPERTILIONIDAE | DD | 0 | 500 | 3063 | 1094 | 762 | 48 | 6% | 1 |
| *Myotis oreias* | VESPERTILIONIDAE | DD | 0 | 9999 | 535 | 532 | 29 | 15 | 51% | 1 |
| *Myotis siligorensis* | VESPERTILIONIDAE | LC | 914 | 2770 | 1103626 | 322948 | 214729 | 35750 | 17% | 0 |
| *Nesolagus timminsi* | LEPORIDAE | DD | 0 | 1000 | 24822 | 21139 | 16782 | 3648 | 22% | 1 |
| *Niviventer brahma* | MURIDAE | LC | 2000 | 2800 | 32328 | 7559 | 6231 | 1503 | 24% | 1 |
| *Niviventer cameroni* | MURIDAE | VU | 1520 | 2020 | 9975 | 562 | 514 | 85 | 16% | 1 |
| *Niviventer langbianis* | MURIDAE | LC | 200 | 1600 | 631984 | 542769 | 359000 | 90710 | 25% | 0 |
| *Niviventer tenaster* | MURIDAE | LC | 1300 | 3000 | 232236 | 21172 | 16130 | 2741 | 17% | 1 |
| *Nomascus concolor* | HYLOBATIDAE | CR | 500 | 2700 | 26326 | 25057 | 17399 | 4614 | 27% | 1 |
| *Nomascus gabriellae* | HYLOBATIDAE | EN | 400 | 1650 | 94846 | 48017 | 33676 | 9743 | 29% | 1 |
| *Nomascus leucogenys* | HYLOBATIDAE | CR | 200 | 1650 | 51342 | 45315 | 28806 | 10180 | 35% | 1 |
| *Nomascus siki* | HYLOBATIDAE | EN | 30 | 1800 | 26549 | 26042 | 20255 | 7724 | 38% | 1 |
| *Nycticebus bengalensis* | LORISIDAE | VU | 0 | 2400 | 1968412 | 1946479 | 828324 | 162762 | 20% | 1 |
| *Nycticebus pygmaeus* | LORISIDAE | VU | 0 | 1500 | 461373 | 449989 | 234911 | 39191 | 17% | 1 |
| *Ochotona gaoligongensis* | OCHOTONIDAE | DD | 2750 | 3250 | 6122 | 1303 | 1004 | 422 | 42% | 1 |
| *Ochotona nigritia* | OCHOTONIDAE | DD | 2950 | 3450 | 2239 | 445 | 341 | 102 | 30% | 1 |
| *Paracoelops megalotis* | HIPPOSIDERIDAE | DD | 0 | 9999 | 17 | 17 | 0 | 0 | 0% | 1 |
| *Phoniscus atrox* | VESPERTILIONIDAE | NT | 0 | 400 | 174557 | 147994 | 42931 | 6799 | 16% | 0 |
| *Pipistrellus anthonyi* | VESPERTILIONIDAE | DD | 1884 | 2384 | 47 | 20 | 15 | 0 | 0% | 1 |
| *Pipistrellus cadornae* | VESPERTILIONIDAE | LC | 250 | 750 | 510460 | 190012 | 120579 | 23638 | 20% | 0 |
| *Pipistrellus joffrei* | VESPERTILIONIDAE | DD | 0 | 1000 | 4014 | 2278 | 2001 | 0 | 0% | 1 |
| *Pipistrellus paterculus* | VESPERTILIONIDAE | LC | 0 | 1500 | 774413 | 612337 | 286201 | 41876 | 15% | 0 |
| *Pithecheir parvus* | MURIDAE | DD | 0 | 1200 | 101355 | 98787 | 44004 | 6834 | 16% | 0 |
| *Presbytis femoralis* | CERCOPITHECIDAE | NT | 0 | 1000 | 126296 | 125538 | 46806 | 14176 | 30% | 0 |
| *Pseudoryx nghetinhensis* | BOVIDAE | CR | 0 | 1200 | 13995 | 12359 | 9534 | 2770 | 29% | 1 |
| *Pteropus lylei* | PTEROPODIDAE | VU | 0 | 1000 | 106247 | 102137 | 11779 | 6076 | 52% | 1 |
| *Pygathrix cinerea* | CERCOPITHECIDAE | CR | 0 | 9999 | 24681 | 24681 | 11361 | 1295 | 11% | 1 |
| *Pygathrix nemaeus* | CERCOPITHECIDAE | EN | 0 | 1600 | 91505 | 90863 | 55781 | 18969 | 34% | 1 |
| *Pygathrix nigripes* | CERCOPITHECIDAE | EN | 0 | 9999 | 95061 | 95061 | 31828 | 5620 | 18% | 1 |
| *Rhinolophus coelophyllus* | RHINOLOPHIDAE | LC | 0 | 1000 | 425270 | 400052 | 127146 | 51693 | 41% | 0 |
| *Rhinolophus convexus* | RHINOLOPHIDAE | DD | 1000 | 9999 | 7651 | 1952 | 1649 | 154 | 9% | 1 |
| *Rhinolophus paradoxolophus* | RHINOLOPHIDAE | LC | 0 | 9999 | 395850 | 395799 | 202021 | 38351 | 19% | 0 |
| *Rhinolophus robinsoni* | RHINOLOPHIDAE | NT | 0 | 1000 | 47257 | 43775 | 16770 | 2266 | 14% | 1 |
| *Rhinolophus shameli* | RHINOLOPHIDAE | LC | 0 | 1000 | 766127 | 678474 | 222464 | 61592 | 28% | 0 |
| *Rhinolophus siamensis* | RHINOLOPHIDAE | LC | 0 | 1000 | 309658 | 209778 | 126596 | 19279 | 15% | 0 |
| *Rhinolophus yunanensis* | RHINOLOPHIDAE | LC | 0 | 1000 | 401193 | 263109 | 155447 | 35078 | 23% | 0 |
| *Rhinopithecus avunculus* | CERCOPITHECIDAE | CR | 200 | 1200 | 10210 | 5912 | 4724 | 95 | 2% | 1 |
| *Rhinopithecus strykeri* | CERCOPITHECIDAE | CR | 2745 | 3660 | 203 | 74 | 51 | 0 | 0% | 1 |
| *Saxatilomys paulinae* | MURIDAE | DD | 0 | 1000 | 2249 | 2240 | 795 | 795 | 100% | 1 |
| *Suncus malayanus* | SORICIDAE | DD | 0 | 1000 | 170649 | 165158 | 61808 | 10990 | 18% | 0 |
| *Sus bucculentus* | SUIDAE | DD | 0 | 9999 | 368 | 368 | 45 | 0 | 0% | 1 |
| *Tadarida johorensis* | MOLOSSIDAE | VU | 0 | 500 | 80625 | 71472 | 29227 | 5079 | 17% | 1 |
| *Taphozous theobaldi* | EMBALLONURIDAE | LC | 0 | 1200 | 998406 | 671650 | 157608 | 58972 | 37% | 0 |
| *Tonkinomys daovantieni* | MURIDAE | DD | 0 | 500 | 117 | 114 | 92 | 92 | 100% | 1 |
| *Trachypithecus barbei* | CERCOPITHECIDAE | DD | 0 | 9999 | 21027 | 21017 | 10952 | 7487 | 68% | 1 |
| *Trachypithecus delacouri* | CERCOPITHECIDAE | CR | 0 | 1000 | 6008 | 5945 | 2106 | 202 | 10% | 1 |
| *Trachypithecus germaini* | CERCOPITHECIDAE | EN | 0 | 1000 | 342521 | 325292 | 95437 | 33752 | 35% | 1 |
| *Trachypithecus hatinhensis* | CERCOPITHECIDAE | EN | 0 | 9999 | 18903 | 18903 | 10563 | 2297 | 22% | 1 |
| *Trachypithecus laotum* | CERCOPITHECIDAE | VU | 1000 | 9999 | 5592 | 706 | 493 | 119 | 24% | 1 |
| *Trachypithecus obscurus* | CERCOPITHECIDAE | NT | 0 | 1000 | 257716 | 251624 | 103545 | 23895 | 23% | 0 |
| *Trachypithecus phayrei* | CERCOPITHECIDAE | EN | 0 | 9999 | 1107063 | 1106443 | 466495 | 75369 | 16% | 1 |
| *Trachypithecus shortridgei* | CERCOPITHECIDAE | EN | 200 | 2500 | 10535 | 5853 | 4906 | 390 | 8% | 1 |
| *Tragulus versicolor* | TRAGULIDAE | DD | 0 | 500 | 1480 | 960 | 409 | 0 | 0% | 1 |
| *Tragulus williamsoni* | TRAGULIDAE | DD | 0 | 9999 | 316 | 316 | 119 | 0 | 0% | 1 |
| *Uropsilus investigator* | TALPIDAE | DD | 3600 | 4600 | 3186 | 803 | 83 | 54 | 64% | 1 |
| *Viverra megaspila* | VIVERRIDAE | VU | 0 | 500 | 1408099 | 944190 | 298597 | 64223 | 22% | 1 |

**Table D. Amphibian Information**

| **Species** | **Family** | **IUCN** | **Elevational Range** | | **Original Range** | **Refined by Elevation** | **Refined by Forest** | **PA** | **PA Coverage** | **Species of Concern** |
| --- | --- | --- | --- | --- | --- | --- | --- | --- | --- | --- |
|  |  |  | **Min/m** | **Max/m** | **km^2^** | **km^2^** | **km^2^** | **km^2^** |  |  |
| *Amolops archotaphus* | RANIDAE | LC | 600 | 1800 | 6359 | 5131 | 4044 | 1289 | 0 | 1 |
| *Amolops assamensis* | RANIDAE | DD | 1000 | 9999 | 1150 | 0 | 0 | 0 |  | 1 |
| *Amolops bellulus* | RANIDAE | DD | 1330 | 1830 | 389 | 32 | 26 | 25 | 1 | 1 |
| *Amolops caelumnoctis* | RANIDAE | DD | 2150 | 2650 | 83 | 30 | 29 | 28 | 1 | 1 |
| *Amolops compotrix* | RANIDAE | DD | 200 | 2000 | 30 | 28 | 25 | 25 | 1 | 1 |
| *Amolops cremnobatus* | RANIDAE | NT | 200 | 1300 | 41826 | 30745 | 24512 | 4370 | 0 | 0 |
| *Amolops cucae* | RANIDAE | DD | 390 | 890 | 25 | 14 | 13 | 0 | 0 | 1 |
| *Amolops iriodes* | RANIDAE | DD | 1300 | 1800 | 9 | 6 | 6 | 0 | 0 | 1 |
| *Amolops kaulbacki* | RANIDAE | DD | 0 | 9999 | 255 | 255 | 193 | 193 | 1 | 1 |
| *Amolops larutensis* | RANIDAE | LC | 43 | 1500 | 104863 | 88572 | 51091 | 8009 | 0 | 0 |
| *Amolops longimanus* | RANIDAE | DD | 1750 | 2250 | 465 | 80 | 65 | 0 | 0 | 1 |
| *Amolops mengyangensis* | RANIDAE | LC | 680 | 1900 | 5391 | 4326 | 1899 | 546 | 0 | 1 |
| *Amolops minutus* | RANIDAE | DD | 2050 | 2400 | 153 | 44 | 40 | 0 | 0 | 1 |
| *Amolops panhai* | RANIDAE | LC | 0 | 500 | 23891 | 17779 | 11277 | 3105 | 0 | 1 |
| *Amolops spinapectoralis* | RANIDAE | DD | 650 | 1150 | 3161 | 1117 | 581 | 65 | 0 | 1 |
| *Amolops splendissimus* | RANIDAE | DD | 1850 | 2400 | 153 | 64 | 57 | 0 | 0 | 1 |
| *Amolops tuberodepressus* | RANIDAE | VU | 1000 | 2400 | 7055 | 6193 | 3420 | 119 | 0 | 1 |
| *Amolops viridimaculatus* | RANIDAE | NT | 1400 | 2350 | 10982 | 4860 | 3351 | 184 | 0 | 1 |
| *Amolops vitreus* | RANIDAE | DD | 0 | 600 | 99 | 9 | 5 | 5 | 1 | 1 |
| *Annandia delacouri* | DICROGLOSSIDAE | DD | 600 | 1100 | 13743 | 2281 | 1555 | 103 | 0 | 1 |
| *Ansonia endauensis* | BUFONIDAE | DD | 0 | 500 | 33 | 30 | 25 | 25 | 1 | 1 |
| *Ansonia inthanon* | BUFONIDAE | DD | 930 | 1650 | 2684 | 1075 | 930 | 279 | 0 | 1 |
| *Ansonia jeetsukumarani* | BUFONIDAE | DD | 842 | 1342 | 3408 | 401 | 382 | 0 | 0 | 1 |
| *Ansonia kraensis* | BUFONIDAE | DD | 0 | 500 | 9120 | 8022 | 4526 | 3314 | 1 | 1 |
| *Ansonia latiffi* | BUFONIDAE | DD | 5 | 505 | 18589 | 15609 | 9559 | 2672 | 0 | 1 |
| *Ansonia latirostra* | BUFONIDAE | DD | 5 | 505 | 121 | 31 | 13 | 0 | 0 | 1 |
| *Ansonia malayana* | BUFONIDAE | LC | 300 | 1300 | 84543 | 28668 | 25591 | 5939 | 0 | 0 |
| *Ansonia siamensis* | BUFONIDAE | VU | 0 | 1300 | 1442 | 1442 | 747 | 683 | 1 | 1 |
| *Ansonia tiomanica* | BUFONIDAE | VU | 300 | 1000 | 45 | 34 | 29 | 29 | 1 | 1 |
| *Brachytarsophrys carinense* | MEGOPHRYIDAE | LC | 500 | 9999 | 134025 | 91606 | 57646 | 18656 | 0 | 0 |
| *Brachytarsophrys feae* | MEGOPHRYIDAE | LC | 650 | 2100 | 305191 | 214475 | 82060 | 4570 | 0 | 0 |
| *Brachytarsophrys intermedia* | MEGOPHRYIDAE | VU | 900 | 9999 | 9524 | 476 | 312 | 18 | 0 | 1 |
| *Bufo ailaoanus* | BUFONIDAE | DD | 2325 | 2825 | 2021 | 6 | 5 | 0 | 0 | 1 |
| *Bufo aspinius* | BUFONIDAE | DD | 1800 | 2400 | 1231 | 475 | 102 | 8 | 0 | 1 |
| *Bufo pageoti* | BUFONIDAE | NT | 1900 | 2500 | 16088 | 2601 | 1821 | 217 | 0 | 1 |
| *Bufoides meghalayanus* | BUFONIDAE | EN | 888 | 1442 | 322 | 9 | 2 | 0 | 0 | 1 |
| *Calluella minuta* | MICROHYLIDAE | DD | 167 | 667 | 20 | 10 | 8 | 8 | 1 | 1 |
| *Chiromantis cherrapunjiae* | RHACOPHORIDAE | DD | 880 | 1380 | 969 | 315 | 154 | 0 | 0 | 1 |
| *Chiromantis hansenae* | RHACOPHORIDAE | DD | 0 | 900 | 49 | 49 | 2 | 2 | 1 | 1 |
| *Chiromantis samkosensis* | RHACOPHORIDAE | DD | 0 | 1000 | 20 | 20 | 20 | 20 | 1 | 1 |
| *Chiromantis shyamrupus* | RHACOPHORIDAE | DD | 250 | 750 | 480 | 49 | 44 | 0 | 0 | 1 |
| *Duttaphrynus crocus* | BUFONIDAE | DD | 0 | 100 | 20 | 5 | 3 | 0 | 0 | 1 |
| *Duttaphrynus stuarti* | BUFONIDAE | DD | 556 | 640 | 10233 | 315 | 277 | 0 | 0 | 1 |
| *Feihyla palpebralis* | RHACOPHORIDAE | NT | 700 | 2000 | 16924 | 9856 | 4846 | 903 | 0 | 1 |
| *Fejervarya raja* | DICROGLOSSIDAE | DD | 0 | 9999 | 2236 | 2232 | 154 | 101 | 1 | 1 |
| *Fejervarya triora* | DICROGLOSSIDAE | DD | 230 | 360 | 15 | 8 | 3 | 3 | 1 | 1 |
| *Gracixalus gracilipes* | RHACOPHORIDAE | LC | 600 | 1800 | 38679 | 26282 | 15653 | 4636 | 0 | 1 |
| *Gracixalus quyeti* | RHACOPHORIDAE | EN | 300 | 1100 | 1394 | 897 | 791 | 279 | 0 | 1 |
| *Gracixalus supercornutus* | RHACOPHORIDAE | DD | 0 | 1300 | 28 | 28 | 25 | 9 | 0 | 1 |
| *Hylarana attigua* | RANIDAE | VU | 0 | 9999 | 13624 | 13624 | 8824 | 2260 | 0 | 1 |
| *Hylarana banjarana* | RANIDAE | NT | 700 | 1300 | 20558 | 7651 | 7100 | 589 | 0 | 1 |
| *Hylarana cubitalis* | RANIDAE | LC | 500 | 760 | 361928 | 87564 | 61715 | 15147 | 0 | 0 |
| *Hylarana laterimaculata* | RANIDAE | LC | 0 | 1000 | 95380 | 90159 | 44404 | 7060 | 0 | 0 |
| *Hylarana leptoglossa* | RANIDAE | LC | 0 | 700 | 66276 | 55560 | 19572 | 1313 | 0 | 1 |
| *Hylarana maosonensis* | RANIDAE | LC | 200 | 1300 | 70839 | 43059 | 31551 | 3805 | 0 | 0 |
| *Hylarana milleti* | RANIDAE | LC | 600 | 1200 | 27690 | 4225 | 3760 | 3522 | 1 | 1 |
| *Hylarana montivaga* | RANIDAE | LC | 700 | 1500 | 15594 | 7216 | 5006 | 1489 | 0 | 1 |
| *Hylarana mortenseni* | RANIDAE | NT | 1 | 800 | 23166 | 21051 | 12828 | 7821 | 1 | 1 |
| *Hylarana nigrovittata* | RANIDAE | LC | 60 | 1200 | 1768060 | 1331043 | 582703 | 125408 | 0 | 0 |
| *Ichthyophis khumhzi* | ICHTHYOPHIIDAE | DD | 137 | 637 | 34 | 21 | 4 | 0 | 0 | 1 |
| *Ichthyophis larutensis* | ICHTHYOPHIIDAE | DD | 500 | 1000 | 754 | 174 | 163 | 33 | 0 | 1 |
| *Ichthyophis supachaii* | ICHTHYOPHIIDAE | DD | 0 | 1000 | 11595 | 11368 | 3360 | 1720 | 1 | 1 |
| *Ichthyophis youngorum* | ICHTHYOPHIIDAE | DD | 1000 | 1200 | 266 | 62 | 55 | 35 | 1 | 1 |
| *Ingerana borealis* | DICROGLOSSIDAE | VU | 0 | 500 | 125714 | 76586 | 15399 | 678 | 0 | 1 |
| *Ingerana liui* | DICROGLOSSIDAE | VU | 550 | 760 | 30284 | 2906 | 565 | 144 | 0 | 1 |
| *Ingerana tasanae* | DICROGLOSSIDAE | VU | 750 | 1250 | 6143 | 1107 | 1069 | 1063 | 1 | 1 |
| *Ingerana tenasserimensis* | DICROGLOSSIDAE | LC | 300 | 600 | 54117 | 14625 | 12106 | 4514 | 0 | 1 |
| *Ingerophrynus galeatus* | BUFONIDAE | LC | 250 | 1300 | 170817 | 118280 | 75133 | 17029 | 0 | 0 |
| *Ingerophrynus gollum* | BUFONIDAE | DD | 0 | 546 | 33 | 31 | 26 | 26 | 1 | 1 |
| *Ingerophrynus kumquat* | BUFONIDAE | EN | 0 | 1000 | 2119 | 2117 | 698 | 23 | 0 | 1 |
| *Kalophrynus palmatissimus* | MICROHYLIDAE | EN | 0 | 500 | 646 | 559 | 361 | 8 | 0 | 1 |
| *Kalophrynus robinsoni* | MICROHYLIDAE | DD | 0 | 9999 | 453 | 453 | 376 | 376 | 1 | 1 |
| *Kaloula aureata* | MICROHYLIDAE | DD | 0 | 1000 | 10696 | 10552 | 2434 | 1635 | 1 | 1 |
| *Kaloula mediolineata* | MICROHYLIDAE | NT | 0 | 1000 | 214351 | 209199 | 22455 | 11600 | 1 | 0 |
| *Kurixalus ananjevae* | RHACOPHORIDAE | DD | 1250 | 1750 | 9 | 0 | 0 | 0 |  | 1 |
| *Kurixalus baliogaster* | RHACOPHORIDAE | VU | 700 | 1000 | 11581 | 3174 | 1646 | 418 | 0 | 1 |
| *Kurixalus banaensis* | RHACOPHORIDAE | DD | 0 | 1000 | 356 | 336 | 147 | 23 | 0 | 1 |
| *Kurixalus bisacculus* | RHACOPHORIDAE | LC | 1450 | 2000 | 81090 | 1231 | 635 | 66 | 0 | 1 |
| *Kurixalus carinensis* | RHACOPHORIDAE | DD | 1250 | 2000 | 5179 | 1048 | 931 | 90 | 0 | 1 |
| *Kurixalus naso* | RHACOPHORIDAE | DD | 1100 | 1500 | 11824 | 2162 | 1539 | 118 | 0 | 1 |
| *Kurixalus verrucosus* | RHACOPHORIDAE | LC | 200 | 1200 | 197388 | 141518 | 94760 | 26208 | 0 | 0 |
| *Laotriton laoensis* | SALAMANDRIDAE | EN | 1045 | 1545 | 4559 | 2967 | 2203 | 0 | 0 | 1 |
| *Leptobrachium ailaonicum* | MEGOPHRYIDAE | NT | 800 | 2600 | 13728 | 13102 | 7315 | 518 | 0 | 1 |
| *Leptobrachium banae* | MEGOPHRYIDAE | VU | 800 | 1600 | 11232 | 6950 | 5461 | 1268 | 0 | 1 |
| *Leptobrachium buchardi* | MEGOPHRYIDAE | DD | 835 | 1335 | 9 | 9 | 6 | 0 | 0 | 1 |
| *Leptobrachium chapaense* | MEGOPHRYIDAE | LC | 800 | 2400 | 412115 | 247082 | 149615 | 23466 | 0 | 0 |
| *Leptobrachium echinatum* | MEGOPHRYIDAE | EN | 1900 | 2500 | 683 | 165 | 131 | 35 | 0 | 1 |
| *Leptobrachium huashen* | MEGOPHRYIDAE | LC | 1750 | 2250 | 143625 | 31993 | 16920 | 727 | 0 | 1 |
| *Leptobrachium mouhoti* | MEGOPHRYIDAE | DD | 500 | 1480 | 692 | 622 | 201 | 84 | 0 | 1 |
| *Leptobrachium ngoclinhense* | MEGOPHRYIDAE | DD | 1500 | 2000 | 58 | 19 | 17 | 17 | 1 | 1 |
| *Leptobrachium nigrops* | MEGOPHRYIDAE | LC | 0 | 500 | 55132 | 48866 | 16028 | 2666 | 0 | 1 |
| *Leptobrachium promustache* | MEGOPHRYIDAE | DD | 2000 | 2500 | 105 | 14 | 14 | 8 | 1 | 1 |
| *Leptobrachium smithi* | MEGOPHRYIDAE | LC | 0 | 1400 | 205922 | 202264 | 89181 | 35997 | 0 | 0 |
| *Leptobrachium xanthospilum* | MEGOPHRYIDAE | DD | 550 | 1050 | 3330 | 1685 | 639 | 85 | 0 | 1 |
| *Leptolalax alpinus* | MEGOPHRYIDAE | EN | 2150 | 2650 | 3053 | 724 | 502 | 126 | 0 | 1 |
| *Leptolalax bourreti* | MEGOPHRYIDAE | DD | 400 | 2550 | 86 | 77 | 56 | 29 | 1 | 1 |
| *Leptolalax fuliginosus* | MEGOPHRYIDAE | DD | 0 | 9999 | 41 | 41 | 23 | 9 | 0 | 1 |
| *Leptolalax heteropus* | MEGOPHRYIDAE | LC | 300 | 1200 | 51041 | 22184 | 19583 | 2565 | 0 | 1 |
| *Leptolalax melanolecus* | MEGOPHRYIDAE | LC | 0 | 9999 | 903 | 903 | 696 | 644 | 1 | 1 |
| *Leptolalax nahangensis* | MEGOPHRYIDAE | DD | 300 | 800 | 550 | 298 | 199 | 3 | 0 | 1 |
| *Leptolalax pelodytoides* | MEGOPHRYIDAE | LC | 200 | 1900 | 640544 | 545341 | 362629 | 80928 | 0 | 0 |
| *Leptolalax pluvialis* | MEGOPHRYIDAE | DD | 1600 | 2100 | 546 | 25 | 22 | 0 | 0 | 1 |
| *Leptolalax sungi* | MEGOPHRYIDAE | DD | 750 | 1250 | 415 | 37 | 34 | 34 | 1 | 1 |
| *Leptolalax tuberosus* | MEGOPHRYIDAE | VU | 800 | 1300 | 3835 | 1907 | 1237 | 218 | 0 | 1 |
| *Leptolalax ventripunctatus* | MEGOPHRYIDAE | DD | 675 | 1175 | 278 | 194 | 92 | 0 | 0 | 1 |
| *Limnonectes dabanus* | DICROGLOSSIDAE | DD | 0 | 700 | 22921 | 11332 | 4440 | 478 | 0 | 1 |
| *Limnonectes doriae* | DICROGLOSSIDAE | DD | 0 | 1100 | 152911 | 148976 | 79081 | 18123 | 0 | 0 |
| *Limnonectes gyldenstolpei* | DICROGLOSSIDAE | LC | 200 | 2200 | 233677 | 184355 | 108492 | 43832 | 0 | 0 |
| *Limnonectes hascheanus* | DICROGLOSSIDAE | LC | 50 | 1000 | 209390 | 164806 | 104399 | 30458 | 0 | 0 |
| *Limnonectes khammonensis* | DICROGLOSSIDAE | DD | 0 | 9999 | 58 | 58 | 52 | 46 | 1 | 1 |
| *Limnonectes macrognathus* | DICROGLOSSIDAE | DD | 1 | 9999 | 55182 | 55173 | 33556 | 7859 | 0 | 0 |
| *Limnonectes mawlyndipi* | DICROGLOSSIDAE | DD | 1250 | 1750 | 125 | 125 | 4 | 0 | 0 | 1 |
| *Limnonectes nitidus* | DICROGLOSSIDAE | EN | 900 | 1500 | 799 | 289 | 279 | 0 | 0 | 1 |
| *Limnonectes plicatellus* | DICROGLOSSIDAE | LC | 1 | 1200 | 94278 | 91566 | 46825 | 7322 | 0 | 0 |
| *Limnonectes poilani* | DICROGLOSSIDAE | LC | 100 | 1000 | 61270 | 52655 | 18471 | 2860 | 0 | 1 |
| *Limnonectes toumanoffi* | DICROGLOSSIDAE | VU | 1 | 900 | 16477 | 15194 | 3611 | 745 | 0 | 1 |
| *Limnonectes tweediei* | DICROGLOSSIDAE | NT | 200 | 900 | 47111 | 21853 | 18197 | 2231 | 0 | 1 |
| *Microhyla annamensis* | MICROHYLIDAE | LC | 600 | 1200 | 78526 | 32794 | 17749 | 3404 | 0 | 1 |
| *Microhyla annectens* | MICROHYLIDAE | DD | 1200 | 1900 | 3458 | 47 | 45 | 28 | 1 | 1 |
| *Microhyla erythropoda* | MICROHYLIDAE | DD | 1 | 9999 | 369 | 369 | 256 | 72 | 0 | 1 |
| *Microhyla fusca* | MICROHYLIDAE | DD | 1 | 9999 | 633 | 633 | 208 | 22 | 0 | 1 |
| *Microhyla mantheyi* | MICROHYLIDAE | LC | 1 | 500 | 49644 | 37077 | 18626 | 3882 | 0 | 1 |
| *Microhyla marmorata* | MICROHYLIDAE | LC | 170 | 1300 | 5465 | 4085 | 3472 | 1491 | 0 | 1 |
| *Microhyla nanapollexa* | MICROHYLIDAE | DD | 1230 | 1730 | 9 | 6 | 6 | 0 | 0 | 1 |
| *Microhyla pulverata* | MICROHYLIDAE | DD | 475 | 975 | 9 | 0 | 0 | 0 |  | 1 |
| *Nanorana aenea* | DICROGLOSSIDAE | DD | 1250 | 1750 | 433 | 2 | 2 | 0 | 0 | 1 |
| *Nanorana bourreti* | DICROGLOSSIDAE | DD | 1570 | 2090 | 2452 | 296 | 238 | 58 | 0 | 1 |
| *Nanorana fansipani* | DICROGLOSSIDAE | DD | 1250 | 2050 | 2268 | 361 | 240 | 65 | 0 | 1 |
| *Nanorana feae* | DICROGLOSSIDAE | DD | 1 | 9999 | 6148 | 6148 | 3791 | 0 | 0 | 1 |
| *Nanorana maculosa* | DICROGLOSSIDAE | EN | 1800 | 2600 | 16428 | 4468 | 3184 | 222 | 0 | 1 |
| *Nanorana mokokchungensis* | DICROGLOSSIDAE | DD | 950 | 1450 | 22 | 17 | 3 | 0 | 0 | 1 |
| *Odorrana absita* | RANIDAE | DD | 860 | 1360 | 102 | 53 | 49 | 49 | 1 | 1 |
| *Odorrana aureola* | RANIDAE | DD | 1100 | 1500 | 820 | 218 | 151 | 151 | 1 | 1 |
| *Odorrana bacboensis* | RANIDAE | DD | 0 | 500 | 214 | 137 | 114 | 36 | 0 | 1 |
| *Odorrana banaorum* | RANIDAE | DD | 550 | 1050 | 661 | 550 | 409 | 125 | 0 | 1 |
| *Odorrana bolavensis* | RANIDAE | DD | 850 | 1350 | 5 | 5 | 5 | 4 | 1 | 1 |
| *Odorrana chapaensis* | RANIDAE | NT | 800 | 1700 | 28550 | 7758 | 4675 | 224 | 0 | 1 |
| *Odorrana gigatympana* | RANIDAE | DD | 950 | 1450 | 23 | 23 | 12 | 0 | 0 | 1 |
| *Odorrana indeprensa* | RANIDAE | DD | 1 | 9999 | 878 | 878 | 796 | 776 | 1 | 1 |
| *Odorrana jingdongensis* | RANIDAE | VU | 1000 | 1900 | 110920 | 75298 | 30637 | 1462 | 0 | 1 |
| *Odorrana khalam* | RANIDAE | DD | 1100 | 1600 | 100 | 40 | 39 | 39 | 1 | 1 |
| *Odorrana livida* | RANIDAE | DD | 200 | 700 | 9 | 8 | 3 | 0 | 0 | 1 |
| *Odorrana melasma* | RANIDAE | DD | 350 | 850 | 98 | 60 | 54 | 51 | 1 | 1 |
| *Odorrana monjerai* | RANIDAE | DD | 470 | 970 | 44 | 30 | 28 | 25 | 1 | 1 |
| *Odorrana morafkai* | RANIDAE | LC | 200 | 1500 | 40778 | 37606 | 25982 | 6487 | 0 | 0 |
| *Odorrana nasica* | RANIDAE | LC | 600 | 1500 | 45304 | 24858 | 19177 | 9828 | 1 | 1 |
| *Odorrana orba* | RANIDAE | DD | 200 | 700 | 59 | 21 | 10 | 5 | 1 | 1 |
| *Odorrana tiannanensis* | RANIDAE | LC | 120 | 1000 | 43232 | 23070 | 13076 | 3466 | 0 | 1 |
| *Odorrana trankieni* | RANIDAE | DD | 1 | 9999 | 42 | 42 | 24 | 0 | 0 | 1 |
| *Ophryophryne gerti* | MEGOPHRYIDAE | DD | 1 | 9999 | 30 | 30 | 14 | 14 | 1 | 1 |
| *Ophryophryne hansi* | MEGOPHRYIDAE | DD | 475 | 975 | 10 | 2 | 1 | 1 | 1 | 1 |
| *Ophryophryne microstoma* | MEGOPHRYIDAE | LC | 220 | 1500 | 190155 | 149610 | 89971 | 13828 | 0 | 0 |
| *Ophryophryne pachyproctus* | MEGOPHRYIDAE | LC | 250 | 1700 | 147316 | 119342 | 73000 | 10039 | 0 | 0 |
| *Ophryophryne synoria* | MEGOPHRYIDAE | DD | 1 | 9999 | 28 | 28 | 22 | 22 | 1 | 1 |
| *Oreolalax granulosus* | MEGOPHRYIDAE | VU | 2125 | 2625 | 610 | 156 | 150 | 68 | 0 | 1 |
| *Oreolalax jingdongensis* | MEGOPHRYIDAE | VU | 1500 | 2450 | 12702 | 7628 | 3235 | 88 | 0 | 1 |
| *Paramesotriton deloustali* | SALAMANDRIDAE | VU | 600 | 1200 | 23506 | 2967 | 2226 | 101 | 0 | 1 |
| *Pedostibes kempi* | BUFONIDAE | DD | 512 | 1012 | 6 | 0 | 0 | 0 |  | 1 |
| *Pelophylax lateralis* | RANIDAE | LC | 60 | 1000 | 1059293 | 778336 | 298477 | 77111 | 0 | 0 |
| *Philautus abditus* | RHACOPHORIDAE | DD | 475 | 975 | 594 | 537 | 329 | 0 | 0 | 1 |
| *Philautus cardamonus* | RHACOPHORIDAE | DD | 1200 | 1700 | 86 | 46 | 44 | 44 | 1 | 1 |
| *Philautus cinerascens* | RHACOPHORIDAE | DD | 0 | 9999 | 807 | 807 | 141 | 8 | 0 | 1 |
| *Philautus garo* | RHACOPHORIDAE | VU | 0 | 500 | 4379 | 2552 | 398 | 0 | 0 | 1 |
| *Philautus kempiae* | RHACOPHORIDAE | DD | 0 | 1000 | 25 | 25 | 3 | 0 | 0 | 1 |
| *Philautus maosonensis* | RHACOPHORIDAE | DD | 700 | 1200 | 978 | 200 | 157 | 47 | 0 | 1 |
| *Philautus microdiscus* | RHACOPHORIDAE | DD | 0 | 500 | 1813 | 243 | 111 | 0 | 0 | 1 |
| *Philautus petilus* | RHACOPHORIDAE | DD | 550 | 650 | 9 | 2 | 2 | 2 | 1 | 1 |
| *Philautus truongsonensis* | RHACOPHORIDAE | DD | 300 | 1400 | 227 | 194 | 177 | 120 | 1 | 1 |
| *Philautus tytthus* | RHACOPHORIDAE | DD | 0 | 9999 | 5852 | 5852 | 5051 | 570 | 0 | 1 |
| *Philautus vermiculatus* | RHACOPHORIDAE | LC | 500 | 1600 | 44904 | 13895 | 12713 | 2021 | 0 | 1 |
| *Pterorana khare* | RANIDAE | VU | 200 | 1600 | 6650 | 5496 | 3038 | 738 | 0 | 1 |
| *Quasipaa fasciculispina* | DICROGLOSSIDAE | VU | 150 | 1000 | 12745 | 9874 | 8273 | 6064 | 1 | 1 |
| *Quasipaa verrucospinosa* | DICROGLOSSIDAE | NT | 500 | 1700 | 56238 | 27321 | 18288 | 2665 | 0 | 1 |
| *Raorchestes sahai* | RHACOPHORIDAE | DD | 0 | 9999 | 13 | 13 | 12 | 0 | 0 | 1 |
| *Raorchestes shillongensis* | RHACOPHORIDAE | CR | 0 | 1400 | 79 | 79 | 20 | 0 | 0 | 1 |
| *Rhacophorus annamensis* | RHACOPHORIDAE | VU | 700 | 1200 | 31478 | 11263 | 5309 | 1044 | 0 | 1 |
| *Rhacophorus calcaneus* | RHACOPHORIDAE | NT | 220 | 1300 | 46603 | 39350 | 23282 | 4671 | 0 | 0 |
| *Rhacophorus dorsoviridis* | RHACOPHORIDAE | DD | 1600 | 2100 | 824 | 231 | 186 | 51 | 0 | 1 |
| *Rhacophorus duboisi* | RHACOPHORIDAE | DD | 1675 | 2175 | 1569 | 146 | 119 | 48 | 0 | 1 |
| *Rhacophorus exechopygus* | RHACOPHORIDAE | VU | 800 | 1400 | 19353 | 9592 | 7471 | 1514 | 0 | 1 |
| *Rhacophorus helenae* | RHACOPHORIDAE | EN | 0 | 500 | 308 | 234 | 145 | 66 | 0 | 1 |
| *Rhacophorus hoanglienensis* | RHACOPHORIDAE | DD | 1100 | 1900 | 4568 | 2199 | 1303 | 0 | 0 | 1 |
| *Rhacophorus jarujini* | RHACOPHORIDAE | DD | 115 | 615 | 292 | 292 | 63 | 5 | 0 | 1 |
| *Rhacophorus kio* | RHACOPHORIDAE | VU | 0 | 1400 | 61807 | 58604 | 29532 | 10850 | 0 | 1 |
| *Rhacophorus notater* | RHACOPHORIDAE | DD | 0 | 500 | 409 | 240 | 82 | 0 | 0 | 1 |
| *Rhacophorus orlovi* | RHACOPHORIDAE | LC | 1 | 850 | 16710 | 16019 | 7420 | 1104 | 0 | 1 |
| *Rhacophorus robinsonii* | RHACOPHORIDAE | DD | 200 | 750 | 2240 | 704 | 614 | 69 | 0 | 1 |
| *Rhacophorus vampyrus* | RHACOPHORIDAE | EN | 1470 | 2004 | 1978 | 427 | 406 | 176 | 0 | 1 |
| *Scutiger gongshanensis* | MEGOPHRYIDAE | VU | 2500 | 3800 | 5149 | 2988 | 1604 | 784 | 0 | 1 |
| *Theloderma andersoni* | RHACOPHORIDAE | LC | 1 | 1400 | 11742 | 6927 | 2807 | 221 | 0 | 1 |
| *Theloderma asperum* | RHACOPHORIDAE | LC | 1 | 1400 | 811579 | 783136 | 407450 | 90785 | 0 | 0 |
| *Theloderma bicolor* | RHACOPHORIDAE | EN | 1200 | 1900 | 585 | 287 | 125 | 69 | 1 | 1 |
| *Theloderma corticale* | RHACOPHORIDAE | DD | 800 | 1300 | 1827 | 60 | 56 | 35 | 1 | 1 |
| *Theloderma gordoni* | RHACOPHORIDAE | LC | 700 | 1300 | 28228 | 10847 | 6241 | 1131 | 0 | 1 |
| *Theloderma nagalandense* | RHACOPHORIDAE | DD | 1171 | 1671 | 77 | 44 | 15 | 0 | 0 | 1 |
| *Theloderma phrynoderma* | RHACOPHORIDAE | DD | 0 | 9999 | 204 | 204 | 191 | 0 | 0 | 1 |
| *Theloderma ryabovi* | RHACOPHORIDAE | DD | 960 | 1460 | 25 | 25 | 17 | 0 | 0 | 1 |
| *Theloderma stellatum* | RHACOPHORIDAE | NT | 50 | 1200 | 17309 | 11056 | 5210 | 4404 | 1 | 1 |
| *Tylototriton vietnamensis* | SALAMANDRIDAE | NT | 25 | 525 | 29055 | 18537 | 10038 | 360 | 0 | 1 |
| *Xenophrys auralensis* | MEGOPHRYIDAE | DD | 500 | 1140 | 293 | 167 | 160 | 160 | 1 | 1 |
| *Xenophrys binchuanensis* | MEGOPHRYIDAE | NT | 1900 | 2800 | 6805 | 3307 | 1043 | 4 | 0 | 1 |
| *Xenophrys daweimontis* | MEGOPHRYIDAE | DD | 1650 | 2150 | 2590 | 603 | 306 | 33 | 0 | 1 |
| *Xenophrys giganticus* | MEGOPHRYIDAE | VU | 1400 | 2400 | 4632 | 3159 | 1270 | 134 | 0 | 1 |
| *Xenophrys glandulosa* | MEGOPHRYIDAE | LC | 1 | 2225 | 80754 | 55359 | 20701 | 1150 | 0 | 0 |
| *Xenophrys jingdongensis* | MEGOPHRYIDAE | LC | 1000 | 2400 | 52202 | 42124 | 15762 | 849 | 0 | 1 |
| *Xenophrys lekaguli* | MEGOPHRYIDAE | DD | 550 | 1050 | 2418 | 1119 | 1085 | 1073 | 1 | 1 |
| *Xenophrys longipes* | MEGOPHRYIDAE | NT | 300 | 1000 | 20928 | 11029 | 9746 | 2214 | 0 | 1 |
| *Xenophrys major* | MEGOPHRYIDAE | LC | 250 | 2500 | 879979 | 769904 | 495384 | 83595 | 0 | 0 |
| *Xenophrys palpebralespinosa* | MEGOPHRYIDAE | LC | 600 | 1800 | 70329 | 35646 | 15259 | 1046 | 0 | 1 |
| *Xenophrys parva* | MEGOPHRYIDAE | LC | 500 | 2500 | 473138 | 342905 | 214170 | 30646 | 0 | 0 |
| *Xenophrys serchhipii* | MEGOPHRYIDAE | DD | 630 | 1130 | 51 | 42 | 15 | 0 | 0 | 1 |
| *Xenophrys wuliangshanensis* | MEGOPHRYIDAE | DD | 200 | 2400 | 2328 | 2328 | 1552 | 6 | 0 | 1 |
| *Xenophrys zunhebotoensis* | MEGOPHRYIDAE | DD | 1465 | 1965 | 68 | 68 | 55 | 0 | 0 | 1 |
